# Tunneling spectroscopy of topological insulator Josephson junctions

**DOI:** 10.1126/sciadv.aeg0687

**Published:** 2026-10-30

**Authors:** Jakob Schluck, Ella Nikodem, Anton Montag, Alexander Ziesen, Mahasweta Bagchi, Fabian Hassler, Yoichi Ando

**Affiliations:** ^1^ Physics Institute II, University of Cologne, Zülpicher Str. 77, 50937 Köln, Germany.; ^2^ Institute for Quantum Information, RWTH Aachen University, 52056 Aachen, Germany.; ^3^ Max Planck Institute for the Science of Light, 91058 Erlangen, Germany.; ^4^ Department of Physics, Friedrich-Alexander-Universität Erlangen-Nürnberg, 91058 Erlangen, Germany.

## Abstract

In the seminal proposal by Fu and Kane, the superconducting proximity effect is used to realize topological superconductivity in the topological surface state (TSS) of a 3D topological insulator (TI). In a Josephson junction made on the TI surface, the spin-momentum locking of the TSS guarantees the existence of spin nondegenerate, perfectly transmitted Andreev modes. These modes lead to gap closing as a function of the superconducting phase difference φ. Here, we report the observation of highly transmissive Andreev modes in a TI Josephson junction, probed via tunnel contacts and φ controlled by a flux loop. Within our experimental resolution, they could indicate a gap closing and reopening. This phenomenon is observed for a wide range of chemical potentials, supporting its TSS origin. Under an applied magnetic field, highly transmissive modes are observed locally, matching theoretical predictions for Josephson vortices in the TSS. Our results support the fundamental role of spin-momentum locking in the Andreev physics in the TSS.

## INTRODUCTION

After more than 100 years from the discovery, superconductors remain a fascinating subject in physics. They become even more interesting when topology comes into play (*1*). A special type of superconductor, called “spinless chiral *p*-wave superconductor,” is a prototypical topological superconductor hosting Majorana zero modes at point defects (*1*, *2*). The Majorana zero modes obey non-Abelian exchange statistics (*3*, *4*), which make them a promising candidate for topological quantum computing (*5*). Because of this exciting prospect for applications, the realization of topological superconductivity and the observation of Majorana zero modes have become major themes in condensed matter physics.

Since a spinless chiral *p*-wave superconductor is not yet found in nature, there have been substantial efforts to effectively realize it in a hybrid system (*6*, *7*). Theoretically, a hybrid of a strongly spin-orbit-coupled material and a conventional superconductor would effectively realize the spinless chiral *p*-wave pairing due to spin-momentum locking. The earliest of such proposals made by Fu and Kane (*8*) in 2008 is based on the topological surface state (TSS) of a three-dimensional (3D) topological insulator (TI) proximitized by a conventional *s*-wave superconductor. However, although the spin-momentum locking is most naturally realized in TIs, the experimental evidence for topological superconductivity obtained so far in this platform is weak and relies on indirect signatures, such as modified Fraunhofer patterns (*9*–*11*) and the microwave response (*12*–*14*) of a Josephson junction made on a TI, or a broad zero-bias conductance peak (ZBCP) observed in the Abrikosov vortex core of a proximitized TI (*15*). The search for Majorana zero modes has been more advanced on 1D semiconducting Rashba nanowires, proximity-coupled to a superconductor, and exposed to a strong magnetic field (*7*); however, despite a remarkable experimental progress (*16*, *17*), the distinction between topological and trivial ZBCPs remains contentious in this 1D platform (*18*–*20*). It was recognized early on (*21*) and emphasized in the recent literature (*22*–*25*) that a direct observation of the closing and reopening of the superconducting (SC) gap, which implies the topological phase transition, is a more robust signature of topological superconductivity and thus Majorana zero modes.

In this work, we turn to the TI platform and report the observation of highly transmissive Andreev modes in a TI Josephson junction, which could facilitate a gap closing and reopening upon changing the phase difference φ across the junction. The spin-momentum locking of the TSS prohibits exact backscattering, and hence the normal reflection is forbidden for a perpendicular mode at the boundary to a superconductor. Such an absence of normal backscattering in turn guarantees perfect Andreev reflection, leading to a pair of perfectly transmitted, nondegenerate Andreev bound states (ABSs) in a short Josephson junction. These perfectly transmitted modes become gapless for φ = π, and the spin nondegeneracy of the TSS makes the electron and hole branches to have opposite parity (*26*). As φ is varied, these nondegenerate modes cross, and the ground-state parity alternates at φ = (2*n* + 1)π with *n* integer (*8*). Since the fermion parity is the ℤ_2_ topological invariant of a 1D superconductor (*2*), the gap closing and reopening correspond to a topological phase transition. It is important to emphasize that the appearance of the perfectly transmitted ABSs is a direct consequence of the spin-momentum locking of the TSS that prohibits backscattering. Therefore, as long as the Josephson effect takes place in the TSS, the topological phase transition as a function of φ is a necessity. Nevertheless, this fundamental property has not been experimentally confirmed so far in TI Josephson junctions.

Note that the Fu-Kane theory is only valid for a constant φ along the junction. When an out-of-plane magnetic field is applied to the junction, rendering φ position dependent (*27*), the gapless 1D mode becomes confined at the core of a Josephson vortex, where the local phase difference is φ = (2*n* + 1)π. Because of this, the gapless 1D mode evolves into a Majorana zero mode that is bound to the point defect formed by the Josephson vortex.

In our experiment, we observe strongly dispersing ABSs by tuning φ across the junction in a SQUID geometry and simultaneously detecting the local density of states (LDOS) at different locations, indicating highly transmissive modes. They are observed over a wide range of chemical potentials and can be interpreted within our experimental resolution as a gap closing and reopening. In addition to the modes with the strongest dispersion, there is a multitude of low-lying excited states, as expected from theory. Our interpretation is not affected by whether a ZBCP is observed or there is just a smooth closing of the gap. We further elucidate the locality of the involved states created by the phase gradient in perpendicular magnetic fields. While there are alternative scenarios that could generate a similar phenomenology, our observations can most naturally be understood as consequences of the spin-momentum locking in the TSS. On the basis of this promising result, we suggest concrete conditions to be realized in future experiments to nail down the topological superconductivity and to generate well-protected Majorana zero modes.

## RESULTS

### Phase-biased TI Josephson junction

The ABS spectrum in superconductor-normal-superconductor (SNS)–type Josephson junctions is commonly studied via tunneling spectroscopy by embedding the junction in a SQUID geometry (*28*–*33*). In particular, Ren *et al.* (*31*) and Fornieri *et al.* (*32*) used this technique to look for a topological phase transition in semiconducting Josephson junctions. They relied on the Zeeman effect due to an in-plane magnetic field to lift the spin degeneracy of the ABS and detect the emerging ZBCPs. Here, we apply this technique to the bulk-insulating TI platform, where the topological phase transition does not require a large magnetic field and can be tuned by the phase alone. A scanning electron microscope (SEM) image of our typical device is shown in Fig. 1A. A Josephson junction is created by a thin film of Al on top of an exfoliated flake of the bulk-insulating TI material BiSbTeSe_2_ (*34*, *35*). The length *L* of the junction is about 50 nm, which is shorter than the surface state coherence length (see text S8), making it a short junction to host a single 1D Andreev mode (*30*). The two sides of the junction are connected by an Al loop, which allows us to accurately tune the phase difference φ by applying a perpendicular magnetic field. This device geometry does not allow us to measure the critical current *I*_c_, but our previous study of *I*_c_ in a similar TI Josephson junction (*36*) confirmed that the supercurrent is primarily flowing through the TSS.

**Fig. 1.**
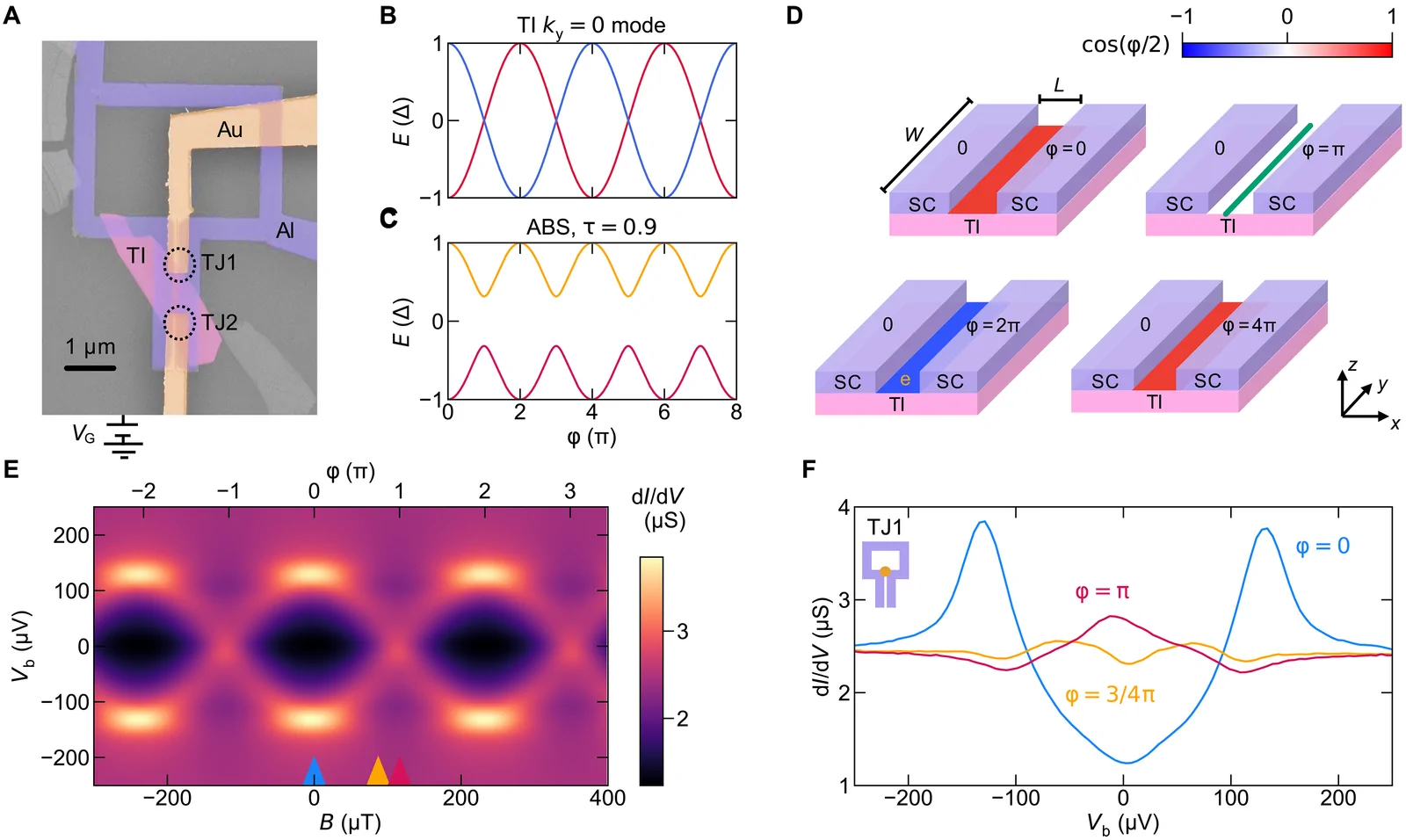
Phase-dependent ABSs in a TI Josephson junction. (**A**) False-color SEM image of a typical device. The exfoliated TI flake (pink) is contacted by Al (lavender) forming an RF-SQUID geometry. Metallic contacts (Au) allow for probing the LDOS at two positions (TJ1 and TJ2) in the junction. (**B** and **C**) Phase dispersions of the perpendicular (*k_y_* = 0) mode in a TI line junction (B) and conventional ABSs with τ = 0.9 (C). In (B), the red and blue lines correspond to the even- and odd-parity bound states, respectively. (**D**) Evolution of the topological phase in the junction; here, the junction area is colored according to the gap function $m\propto \text{cos}\frac{\varphi \left(y\right)}{2}$ (see the color scale). As φ is tuned, the nature of the gap changes from trivial (φ = 0, red) to topological (φ = 2π, blue). This is accompanied by a change in ground-state parity from even to odd, as indicated by the extra electron (yellow) in the junction. In-between, the gap has to close (φ = π) via a gapless Majorana mode (green line). As the phase difference reaches φ = 4π, a full period is completed. (**E**) Color mapping of d*I*/d*V* in the *B* versus *V*_b_ plane measured on TJ1 at 50 mK with *V*_G_ = 0 V. At φ = 0 (mod 2π), a proximity-induced gap opens with conductance peaks at ±130 μV. The gap closes and a broad ZBCP is observed at φ = π. A periodicity of 230 μT is observed, consistent with the device geometry. (**F**) Line cuts of the data in (E) at three selected magnetic fields corresponding to φ = 0, $\frac{3}{4}\pi$, and π.

Normal electrodes made of Au are deposited after putting a thin layer of Al_2_O_3_ as a tunnel barrier. Because of the native oxide of Al, its surface can be treated as an insulator, and the overlap to the Au electrodes does not contribute to the transport. A back-gate voltage can be applied via the degenerately doped silicon substrate. Therefore, this setup enables us to directly probe the LDOS of the TI Josephson junction and to test the Fu-Kane theory (*8*).

The perpendicular mode of the ABSs (i.e., zero longitudinal momentum *k_y_*) appearing in a line junction made on the TSS is expected to present a 4π-periodic φ dependence as shown in Fig. 1B in the short-junction limit. As mentioned above, this is dictated by the spin-momentum locking which prohibits backscattering. The corresponding evolution of the gap in the junction is depicted in Fig. 1D: The gap is trivial for 0 ≤ φ < π. It closes at the topological phase transition with φ = π where the gapless 1D mode (green line) appears, before reopening in the topological regime, with π < φ < 3π. In the topological regime, the parity of the ground state is odd, meaning that the bound state below the chemical potential μ is filled with an unpaired electron (shown in yellow). Because of this, the junction returns to the initial state only at φ = 4π. In contrast, for a conventional ABS, a 2π periodicity is expected, as depicted in Fig. 1C. Here, the dispersion is given by (*37*)

$$E=\pm \Delta \sqrt{1-\tau {\text{sin}}^{2}\left(\varphi /2\right)}$$ (1)

with τ the transmittance of the state and Δ the induced SC gap in the normal metal beneath the SC electrodes. In such a scenario, the gap can close in the case of perfect transmission (τ = 1), but the ground-state parity remains even across the gap closing, as conventional ABSs are doubly degenerate.

Note that experimentally, when the perpendicular magnetic field is very weak such that the large loop is threaded by only a few flux quanta (and no Josephson vortex nucleates in the junction), the phase φ is almost constant along the junction, a situation akin to the Fu-Kane model. The case at larger magnetic field, when Josephson vortices nucleate in the junction, is discussed later.

We focus on the results obtained from a device similar to the one shown in Fig. 1A with two independent tunnel junctions TJ1 and TJ2 located at different positions on the same Josephson junction. Additional data obtained from a second, essentially identical device are shown in text S10 to demonstrate the reproducibility of our results.

### Tunnel spectra

In Fig. 1E, we show the continuous evolution of the differential conductance d*I*/d*V* of TJ1 with the applied dc bias voltage *V*_b_ and the perpendicular magnetic field *B* around zero. Line cuts for selected fields are shown in Fig. 1F. Without magnetic field applied, we observe sharp particle-hole symmetric peaks at the bias voltages of ±130 μeV and a strong reduction in d*I*/d*V* at lower bias. The constant d*I*/d*V* at higher bias corresponds to a resistance of about 400 kohms, indicating that we are in the tunneling regime, where the observed d*I*/d*V* is proportional to the LDOS. The peak energy of 130 μeV is smaller than the SC gap of our Al film, which can be calculated from the critical temperature to be 170 μeV (see text S1). In a different device without TI but conditions that allowed for probing the Al gap directly, we found a density of states consistent with that of a typical BCS superconductor (see text S2). This confirms that the LDOS of the proximitized TI surface, not the LDOS of the Al electrode, is being measured. The LDOS at low bias does not drop to zero but remains finite, which indicates additional in-gap states that are not fully proximitized, consistent with our previous result on similar TI Josephson junctions, where the average transparency was τ*_av_* ≈ 0.8 (*36*). Such a soft gap has also been reported for comparable devices based on carbon nanotubes (*29*), graphene (*30*), HgTe (*31*), or InAs (*32*). Its most likely origin are channels with long quasi-classical trajectories that only couple weakly to the superconductors and can thus have low-lying energy levels with weak phase tunability (*30*, *31*). Since for such channels the short junction approximation is no longer valid, their existence is not captured by models not considering the dynamical phase buildup as in (*8*, *26*). However, it is well known that ballistic proximity systems, if they are extended enough, as well as diffusive ones, if they are disordered enough, will not present a hard induced gap (*38*). In the ballistic case, this effect can be attenuated by specifically designing the device to cut off long trajectories, as was proposed in (*39*).

As a finite phase bias φ is generated across the junction with the application of an out-of-plane magnetic field, the positions of the particle-hole symmetric peaks shift to a lower bias voltage, and their amplitude is diminished. For the magnetic field of about 115 μT that corresponds to φ = π, the particle-hole symmetric peaks merge into a single broad peak at zero bias, signaling the closing of the gap. The spectrum then evolves periodically, with the conductance at 230 μT becoming identical to the one observed at zero field. Having the Fu-Kane theory in mind, our data are consistent with the interpretation that the peak positions trace the gap, corresponding to the lowest-lying state at zero momentum, which evolves with φ and closes at π (see Fig. 1B). In the brighter regions above this gap (but still below the bulk gap), there is a continuum of states corresponding to finite momentum *k_y_* along the junction (*8*), which is reflected in our LDOS spectra. Within this interpretation, the gap closing at φ = π corresponds to a topological phase transition (*8*). Upon close inspection, a certain degree of asymmetry in the LDOS between positive and negative energies can be found in the tunneling conductance. This is a typical phenomenon in similar devices (*30*–*32*), and among the proposed mechanisms behind this effect are nonequilibrium effects (*40*), subgap states in the superconductor (*41*), and voltage-dependent tunnel barriers (*42*). Also, the matrix elements describing the coupling between the normal probe and the ABS are unknown. It is useful to note that similar devices made on various materials in the ballistic regime (*29*, *31*, *32*) with τ < 1 exhibited gapped spectra at φ = π, consistent with Eq. 1. The apparent gap closing observed here is most likely due to the unique property of the TSS, which necessitates the existence of a τ = 1 mode.

To further corroborate this interpretation, we have performed numerical simulations of the differential conductance *G* for a low-energy effective model of a TI Josephson junction (see Materials and Methods for details). Note that we do not aim to accurately model the experimental situation but rather want to demonstrate how they agree with predictions from a rather minimal model. Figure 2A shows the calculated *G* as a function of *V*_b_ and φ, while the line cuts for the same φ values as in Fig. 1F are shown in Fig. 2B. These simulations confirm that the even- and odd-parity bound states having a 4π periodicity produce the two branches that cross at φ = (2*n* + 1)π, as observed in experiments. In Fig. 2B, the coherence peaks show up at the edge of the excitation gap, and they diminish rapidly with increasing φ. Given the limited size of the simulated system, the bulk states above the gap are not correctly captured. Thus, we emphasize that the simulation is only accurate at low energies, below 0.5Δ, and that the position of the coherence peak cannot be traced to larger energies. Our simulations suggest that the exact shape of the spectrum at φ = π depends sensitively on the experimental conditions, and the gap closing can result in a nearly flat d*I*/d*V* (see text S14). All these results are consistent with our experimental observations. It should be emphasized that the gap closing always occurs at φ = (2*n* + 1)π, but it does not need to be accompanied by a ZBCP, according to our simulations.

**Fig. 2.**
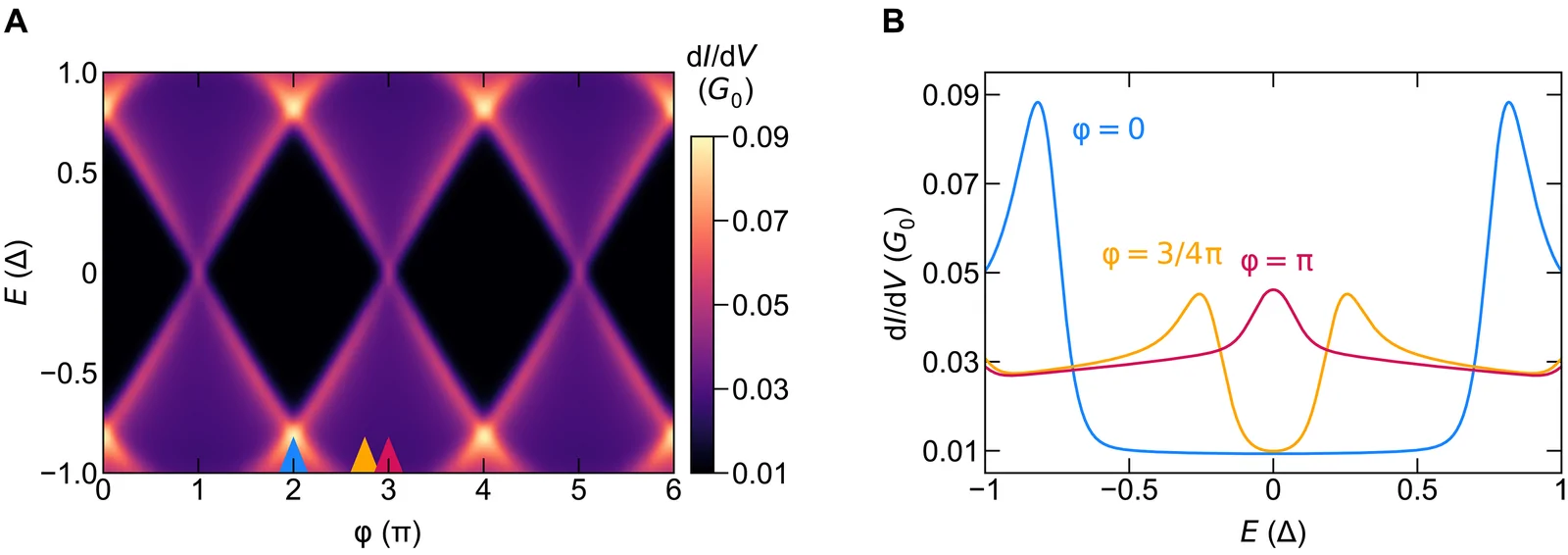
Theoretical simulations. (**A**) Color mapping of the simulated differential conductance d*I*/d*V* in units of the conductance quantum *G*_0_ = *e*^2^/*h* as a function of *V*_b_ and φ for a temperature *T* = Δ/(30*k_B_*) in the Fu-Kane regime with φ constant along the junction. (**B**) Simulated behavior of the differential conductance d*I*/d*V* at φ = 0, $\frac{3}{4}\pi$, and π, which correspond to those shown in Fig. 1F. Note that, in the simulation, only the surface states and a single transversal mode are taken into account, which explains the softer gap in the experimental results of Fig. 1.

### Locality of the spectra in perpendicular magnetic fields

Next, we study the behavior of our device in higher magnetic fields and compare the LDOS probed at TJ1 and TJ2. When a perpendicular magnetic field *B* is applied to the junction, φ becomes dependent on the position along the junction (*y* direction). This locality is reflected in the bound-state spectrum, as recently demonstrated in a semiconductor 2DEG-based junction (*43*). If *B* is strong enough to nucleate Josephson vortices, the position where the local phase difference becomes φ = (2*n* + 1)π is identified as the Josephson vortex core, and in TI Josephson junctions, the gapless 1D mode in the Fu-Kane theory for global φ = (2*n* + 1)π turns into a Majorana zero mode confined to the Josephson vortex core (*27*). The concept of this Majorana zero mode generation is sketched in Fig. 3A. A spatially varying phase φ along a junction caused by *B* leads to a position-dependent gap function $m\propto \text{cos}\frac{\varphi \left(y\right)}{2}$ as indicated by the color code (*27*). Associated with the sign change in the gap function, the gap closes locally at φ(*y*) = (2*n* + 1)π (white area), corresponding to the core of the Josephson vortex and a Majorana zero mode (green dots) nucleates that is confined along the junction by the phase gradient. We note that the topologically protected nature of the gap closing is intuitively understandable in this situation: The region of the junction with −π < φ(*y*) < π (red) has the even-parity ground state with ℤ_2_ index of 0, whereas the region with π < φ(*y*) < 3π (blue) has the odd-parity ground state with ℤ_2_ index of 1; therefore, the boundary between the two regions must become gapless for the topological invariant to change.

**Fig. 3.**
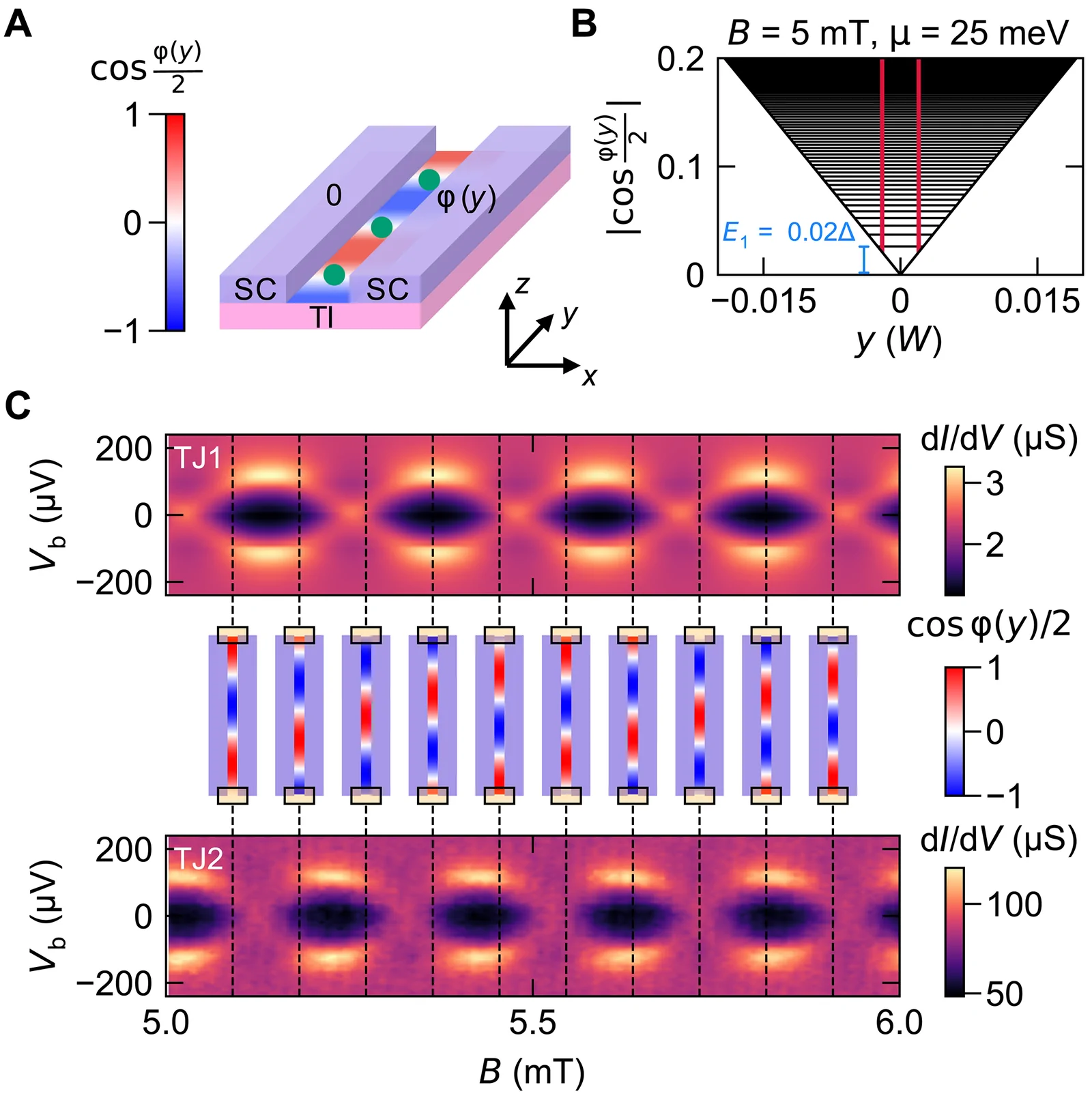
Locality of the spectra. (**A**) Schematic drawing of a planar TI Josephson junction in a perpendicular magnetic field, which generates a position-dependent phase φ(*y*) across the junction. The distribution of the topological gap function $m\propto \text{cos}\frac{\varphi \left(y\right)}{2}$ is depicted in the junction with a color scale. The position of φ(*y*) = (2*n* + 1)π corresponds to the Josephson vortex core, where the gap closes (white color) and a Majorana zero mode (green dot) appears. (**B**) Position-dependent gap $|m|=|\text{cos}\frac{\varphi \left(y\right)}{2}|$ near a Josephson vortex core in a magnetic field of *B* = 5 mT, plotted with the excited states calculated from the model of (*27*) for a chemical potential μ = 25 meV and Δ = 0.17 meV (thin horizontal lines), which form a quasi-continuum with a minigap of only 2% of Δ. The vertical red lines mark the spatial extent of the expected Majorana zero mode appearing around *y* = 0. (**C**) Color mapping of d*I*/d*V* in the *B* versus *V*_b_ plane for TJ1 (top) and TJ2 (bottom) measured in the magnetic field range of 5 to 6 mT at 50 mK with *V*_G_ = −5 V. Note the slight difference in the periodicity, which is 230 μT for TJ1 and 200 μT for TJ2. The middle depicts the expected distribution of the topological gap function in a TI Josephson junction, based on our device geometry, calculated with Eq. 2 that evolves with magnetic fields; the position where the gap function locally becomes zero with φ(*y*) = (2*n* + 1)π corresponds to local gap closing (white regions).

We observed the locality of the ABS spectra, reacting to the local phase difference in a finite magnetic field. The top and bottom of Fig. 3C show the d*I*/d*V* data simultaneously measured at TJ1 and TJ2 as a function of *V*_b_ and *B* in the 5- to 6-mT range. Both junctions present a comparable evolution of their bound state spectrum, but there is a clear phase shift and their periods are slightly different. For TJ2, we find a periodicity of 200 μT, smaller than that of TJ1 (230 μT). This difference in the period is consistent with the sample geometry (see text S3 for details) in which the effective junction area considering the flux focusing effect (*36*, *43*, *44*) is about ∼10% of the loop area. To understand the LDOS at TJ1 and TJ2, we calculated the expected texture of the gap function of a TSS based on the device geometry. In our junctions, the self-inductance is negligible, and their width is much shorter than the Josephson penetration depth (see texts S6 and S7), justifying the description of the phase gradient in terms of

$$\varphi \left(y\right)=\frac{2\pi B}{\Phi_{0}}\left(A+{\mathrm{yL}}_{\text{eff}}\right)$$ (2)

with *A* the loop area, $\Phi_{0}=\frac{h}{2e}$ the magnetic flux quantum, and *L*_eff_ the effective length of the junction taking the flux-focusing effect into account. The evolution of the gap function $m\propto \text{cos}\frac{\varphi \left(y\right)}{2}$ is calculated from this formula with the loop and junction area as fitting parameters (see text S3) and visualized in the middle of Fig. 3C. We show snapshots of the phase texture in the junction for a range of magnetic fields corresponding to the experimental situation. In this range, there are two to three Josephson vortices inside the junction, whose position is tuned by the global perpendicular magnetic field. They enter the junction from the bottom where TJ2 is located and move upward to TJ1 with increasing magnetic field. Whenever a Josephson vortex core crosses the location of a tunnel junction, the energy gap appears to close. This is in line with the local nature of the gap closing predicted in the presence of Josephson vortices. Note that in a similar experiment on a nontopological Josephson junction (*43*), the gap was found to remain open at the Josephson vortex core. Our data shown in Fig. 3C were taken at an applied back-gate voltage of *V*_G_ = −5 V, and we chose the magnetic field range of 5 to 6 mT for the visibility of the phase gradient. The same pattern was found to continue into higher magnetic fields until the Al superconductivity was suppressed at 15 mT (see text S4).

We note that the spectra shown in Fig. 3C essentially look the same as those in Fig. 1E. This is because the Majorana zero mode in the Josephson vortex core is not well separated in energy. For the conditions realized, it is accompanied by a quasi-continuum of low-energy bound states (*27*). Taking the smearing due to the finite temperature into account, the local spectrum becomes a broad peak, indistinguishable from the Fu-Kane situation. In Fig. 3B, we show the magnitude of the gap function near a Josephson vortex core and the finite-energy bound states calculated from the model in (*27*), along with the spatial extent of the expected Majorana zero mode. Assuming a chemical potential of μ = 25 meV that is not very close to the Dirac point, as is the case in our device, the calculated finite-energy bound states form a quasi-continuum with a minigap of only 2% of Δ (i.e., 3 μeV), which is of the order of the thermal energy at the base temperature. For higher chemical potentials, this energy separation is expected to shrink further.

### Stability of the spectra against gating

In the next step, we check the robustness of the observed features with respect to the chemical potential, as tuned by a global back gate. Figure 4A shows the d*I*/d*V* data in *B* = 0 T at a high bias voltage (*V*_b_ = 1 mV) as a function of the back-gate voltage *V*_G_ for both TJ1 and TJ2. The overall tendency is that the conductance decreases for more negative gate voltages, indicating that the TSS is in the *n*-type regime. Superimposed to this general trend, we observe broad peaks (typical peak width of a few volts), which are commonly found in dielectric-based tunneling devices and conventionally explained by resonant states in the dielectric (*30*). We can use their dispersion to estimate the relation between *V*_G_ and μ (see text S5) and find Δμ/Δ*V*_G_ ≈ 1 to 2 meV/V in the given range of chemical potentials, consistent with results reported in our earlier work (*36*). This implies that the chemical potential μ changes by at least 40 meV in the gating range studied here.

**Fig. 4.**
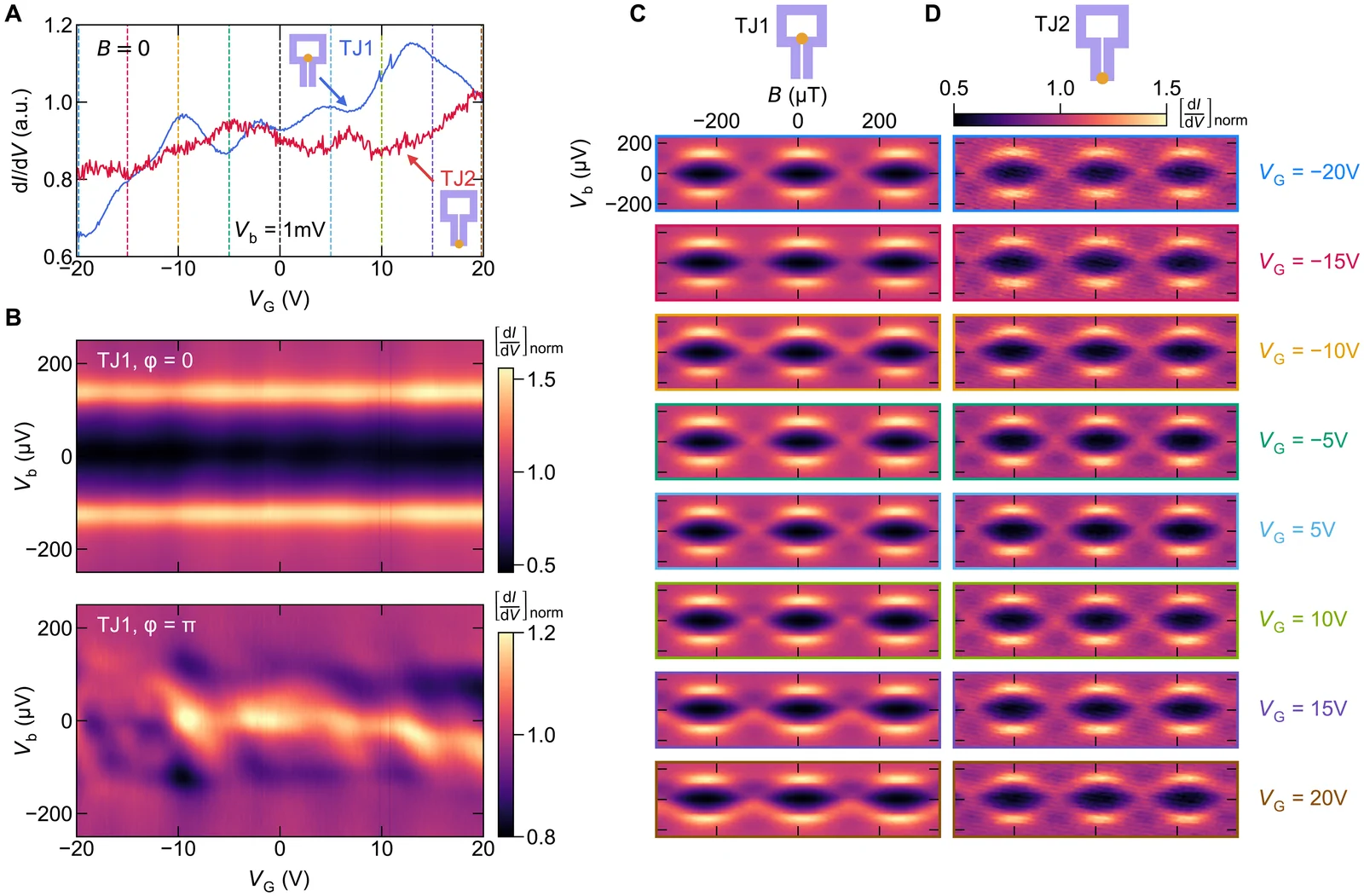
Stability of the spectra against a change in the chemical potential. (**A**) Differential conductance at a high bias voltage of 1 mV as a function of globally applied back-gate voltage *V*_G_. For both tunnel contacts TJ1 and TJ2, the overall conductance is reduced for more negative gate voltages, indicating an *n*-type regime. The vertical dashed lines denote the gate voltages for which the continuous evolution of the spectrum is shown in (C) and (D). (**B**) Normalized d*I*/d*V*, denoted ${\left[\frac{dI}{dV}\right]}_{\text{norm}}$, for TJ1 mapped in the *V*_b_ versus *V*_G_ plane for φ = 0 (top) and φ = π (bottom). The normalization was done at each *V*_G_ by using the d*I*/d*V* value at *V*_b_ = 0.3 mV as reference. (**C** and **D**) Evolution of the *B* dependence of the spectrum for a wide range of *V*_G_ from −20 to +20 V for TJ1 (C) and TJ2 (D). The *V*_G_ values selected for the presentation are equally spaced.

Figure 4B presents the *V*_G_ dependencies of the conductance spectra obtained at TJ1, normalized at each *V*_G_ by using the d*I*/d*V* value at *V*_b_ = 0.3 mV as reference. Here, we show the evolutions of the maximum excitation gap for φ = 0 (top) and the spectrum observed for φ = π (bottom) in a wide range of *V*_G_ from −20 to +20 V. The maximum excitation gap is very stable, indicating that the resonance states due to impurities are not affecting the proximity-induced gap. The apparent gap closing at φ = π is also very stable and is observed at all *V*_G_ values. Note that the occurrence of the broad ZBCP and the details of the LDOS near zero bias appear to depend on *V*_G_, but as we noted above, these features are expected to be sensitive to the details of the experimental conditions; for example, the d*I*/d*V* peak is slightly shifted to the negative *V*_b_ side at *V*_G_ ≈ 12 to 20 V, which is probably due to the strong resonance state located around 13 V (cf. Fig. 4A) crossing the gap energy. We speculate that this strongly breaks particle-hole symmetry, resulting in the shift of the peak from zero to finite energy. What is important in these data is the robust appearance of highly transparent ABSs, potentially indicating a gap closing, which is observed for the whole range of *V*_G_.

In Fig. 4 (C and D), we show the *B* dependencies of the normalized conductance spectra for equally spaced *V*_G_ values between −20 and +20 V for both TJ1 and TJ2. One can immediately see that the basic magnetic field periodic change in d*I*/d*V* is very stable at all *V*_G_ values. Upon close inspection, the breaking of exact electron-hole symmetrical conductance, as mentioned above, is found to be modified by the back-gate voltage. Similar behavior has been reported for comparable devices, e.g., based on graphene or semiconductors (*30*, *32*). Overall, we conclude that the appearance of modes with high transmission is observed irrespective of changes of the chemical potential, possibly indicating the TSS origin. This is further supported by a device with a wider range of gate tunability, showing the same phenomenology (see text S11).

## DISCUSSION

Whether our observations manifest a gap closing and reopening of the proximitized TSS and therefore demonstrate a topological phase transition is the crucial question for the interpretation of our experimental data. From spectroscopy of individual ABSs (see below and text S12), we can estimate the energy resolution of our setup to be about 30 μeV. According to Eq. 1, when Δ ≈ 130 μeV and the experimental energy resolution is 30 μeV, a spectrum with a mini gap would become indistinguishable from a gapless spectrum for τ ≥ 0.95. This places a lower bound on the transmission value that is required to observe an apparent gap closing in our experimental setup. The fact that we consistently observe signatures of such highly transmissive modes makes an interpretation in terms of topologically protected τ = 1 modes plausible but not inevitable. In the following, we also want to discuss topologically trivial scenarios that could result in an apparent gap closing at φ = π. A quantum dot state, symmetrically coupled to SC electrodes and tuned to resonance, leads to a zero-energy state (*45*). This highly fine-tuned case was observed by us in a device where the tunnel barrier was not in a favorable condition to probe the TSS. We found strongly dispersing bound states in a very narrow range of *V*_G_ associated with a spurious ZBCP occurring at a particular *V*_G_ value (see text S12). These features are consistent with the Andreev quantum dot states (*45*) which might occur in the dielectric. The robust spectra discussed in the previous section are clearly of different origin. The spurious ZBCP in this case also tells us that one should not look for a ZBCP but should focus on the gap closing to elucidate the robustness of the phenomenon.

In the literature, the gap closing at φ = π has been reported for diffusive long SNS junctions (*28*, *46*). In such a system, disorder localizes most channels, but it also leads to the appearance of a few high-transmission channels (*47*), which can be responsible for a gap closing.

In the case of our junctions, the mean-free path *l*_e_ ≈ 40 nm has been estimated for the related material of bulk-insulating Bi_1.5_SB_0.5_Te_1.7_Se_1.3_ crystals (*48*), which is a bit shorter but comparable to our junction length (50 nm), putting our device in a quasi-ballistic regime. (Remember that our device is in the short-junction limit where the Fu-Kane theory is applicable.) It is possible that some nearly longitudinal Andreev modes with large *k_y_* traveling for a long distance present the gap closing phenomenology of the diffusive scenario, although we consider it unlikely that this should be as robust against gating as we observe, in particular, as the number of modes in our TI device should be substantially smaller compared to previous experiments on normal metals like Ag (*28*).

On the other hand, even if a more refined experiment would find a finite gap in the spectra, this would not definitely rule out the existence of the topologically protected τ = 1 mode. As has recently been discussed (*49*), in realistic device geometries, the coupling of the modes at the boundary to the ones in the junctions can lift the energies from zero. Similarly, if the wave functions of the ABSs underneath the SC electrons do not decay rapidly enough, they could hybridize with TI states outside of the proximitized region. This effect could be overcome by choosing a different device geometry like the trijunction geometry (*8*) or the nano-SQUID recently proposed by us (*50*).

To make more definite statements about the τ values of individual states and to decide whether a gap closing takes place, using high-frequency measurements to achieve a higher resolution (*51*) would be useful. In this context, it will also be important to obtain better control over the chemical potential in the TI, to control the number of modes in the junction and exclude trivial scenarios. Furthermore, using a charge-sensing technique (*52*) to detect the parity change in the junction before and after the gap closing would unambiguously prove the topological phase transition.

A definitive proof of Majorana zero modes is the demonstration of their non-Abelian exchange statistics through braiding. In a TI Josephson junction, well-protected Majorana zero modes can, in principle, be generated in Josephson vortex cores as we discuss below. Once such protected Majorana zero modes are generated, they can be physically moved along the junction in a controlled manner (*53*); this degree of freedom allows us to conceive braiding operations in a three-slit Josephson junction (*53*), and the resulting change in the parity can be detected by charge sensing (*52*). Another possibility to confirm the non-Abelian statistics is to build a Josephson vortex interferometer proposed by Grosfeld and Stern (*54*), which detects the result of braiding using the Aharonov-Casher effect.

Let us estimate the necessary parameter range to obtain well-protected Majorana zero modes in Josephson vortex cores. There are two quantities that are crucial for this consideration: One is the spatial extent of the Majorana zero mode $\lambda_{B}=\sqrt{v_{M}l_{B}/\pi \Delta }$, where *l*_B_ = Φ_0_/(*BL*_eff_) is the distance between Josephson vortices in the junction and *v*_M_ is the renormalized Majorana velocity. To calculate *v*_M_, we use the formula (see text S13)

$$v_{M}=v_{F}\left(\frac{\Delta^{2}}{\Delta^{2}+\mu^{2}}\right)\frac{\text{cos}\left(\frac{\mu L}{\hbar v_{F}}\right)+\frac{\Delta }{\mu }\text{sin}\left(\frac{\mu L}{\hbar v_{F}}\right)}{1+\frac{\Delta L}{\hbar v_{F}}}$$ (3)

valid in the short-junction regime $L≲\hbar v_{F}/\Delta$ (*L* is the true length of the junction). For large μ >> Δ, the formula goes over to the simple expression $v_{M}≃v_{F}{\left(\Delta /\mu \right)}^{2}$ given in (*27*). The other important quantity is the energy of the first excited state, which is given as $E_{1}\approx \sqrt{2\pi v_{M}\Delta /l_{B}}$ near φ = (2*n* + 1)π (*27*). Note that reducing μ enhances *v*_M_ and thus *E*_1_ and λ_B_. However, λ_B_ must be shorter than *l*_B_/2 to avoid the overlap of neighboring Majorana zero modes which bounds μ from below. To see this competition, we theoretically consider the case of using Nb for the SC electrodes, since its higher magnetic field tolerance allows us to study a wider parameter range as compared to Al. We calculate the dependencies of λ_B_ and *E*_1_ on μ and *B*. The results, shown in Fig. 5 (A and B), indicate that a moderately large μ is helpful to realize a short λ_B_, and the magnetic field helps to obtain a usefully large *E*_1_. In Fig. 5B, we draw the boundaries to guarantee a sufficient separation of Majorana zero modes (λ_B_/*l*_B_ < 0.2, dash-dotted line) and a sufficiently large excitation gap (*E*_1_/Δ > 0.2, solid line). In the region between the lines, see Fig. 5B, Majorana zero modes are well protected. These estimates suggest that it is not necessary to tune μ very close to the Dirac point as long as a magnetic field of the order of 100 mT is used to generate Josephson vortices—an encouraging result for this platform. Possible complications, however, could arise from local variations of the chemical potential as well as Abrikosov vortices generated in the SC electrodes at these magnetic field scales, possibly resulting in unstable phase profiles and magnetic field distributions. Furthermore, it is prudent to note that the soft gap, which we observed in our devices, is a problem for braiding, and a hard gap must be realized by improving the interface transparency between the TI and a superconductor and potentially optimizing the device geometry to eliminate long trajectories (*39*).

**Fig. 5.**
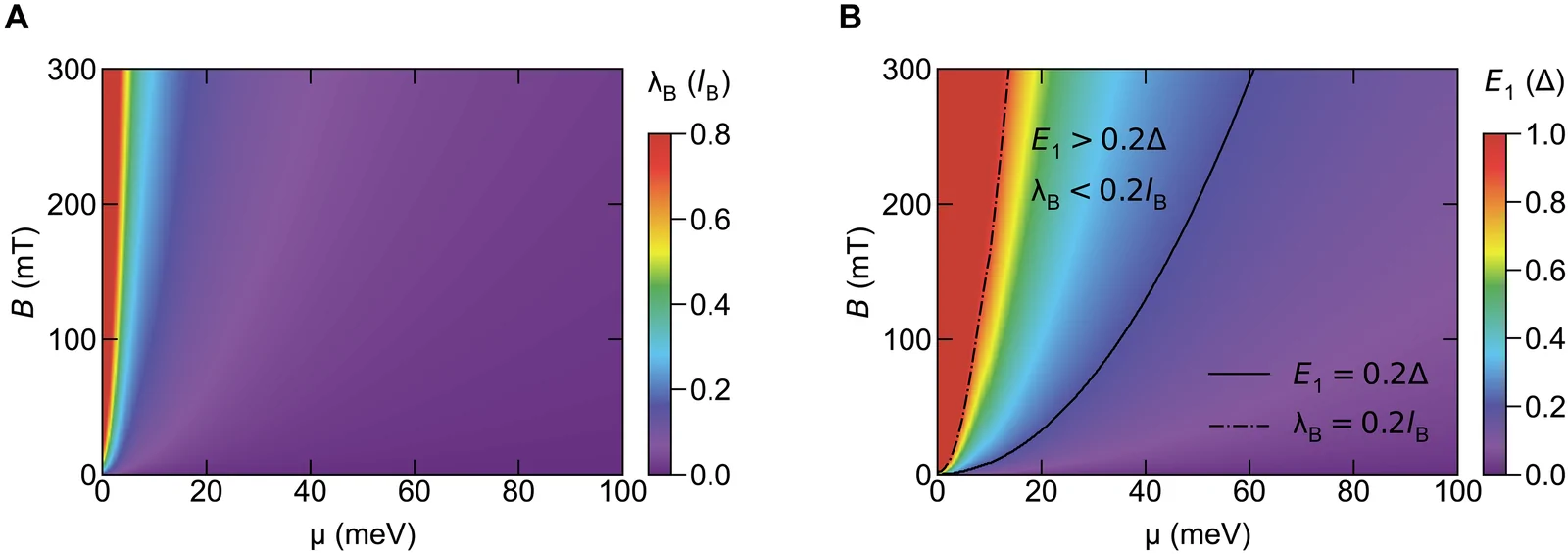
Stability of the Majorana zero modes in the putative case with Nb electrodes. (**A** and **B**) Calculated dependencies of the Majorana bound state width λ_B_ and the first excited state energy *E*_1_ on the chemical potential μ and the perpendicular magnetic field *B* for the Majorana zero mode generated at the Josephson vortex core in a putative device with Nb as the SC electrodes, which will sustain high magnetic fields. We used *L* = 50 nm, *v*_F_ = 5.5 × 10^5^ m/s, and Δ = 1 meV. The dashed and solid black lines in (B) are the boundaries to guarantee a sufficient separation of Majorana zero modes [λ_B_/*l*_B_ < 0.2 obtained from (A)] and a sufficiently large excitation gap (*E*_1_/Δ > 0.2), respectively. Majorana zero modes are well protected in the relatively broad region between the dashed and solid black lines.

In conclusion, we have observed a periodic modulation of the density of states in a phase-biased TI Josephson junction, which could indicate the periodic gap closing and reopening expected from the existence of perfectly transmitted Andreev modes guaranteed by the spin-momentum locking of the TSS. This phenomenon is consistently observed in several devices as a function of an extended parameter range in the chemical potential and the perpendicular magnetic field, which support its TSS origin. The spin nondegeneracy of the TSS would make this a topological phase transition accompanied by a parity change, which remains to be confirmed by future experiments. In the magnetic field range where a couple of Josephson vortices nucleate in the junction, we observed the locality of the spectra implying a confinement of the zero-energy mode (*27*). However, within the magnetic field range where Al remains SC, an abundance of low-lying excited states arises, and this leads to spectra that are indistinguishable from those near zero field. We have discussed the necessary parameter range to realize well-protected Majorana zero modes in the Josephson vortex core. We found that maintaining a chemical potential μ not too close to the Dirac point is crucial for avoiding Majorana delocalization; a μ of approximately 25 meV facilitates the realization of well-protected Majorana zero modes in a magnetic field of 100 mT accessible with a Nb electrode. Considering the high controllability of the Josephson vortex, this result is promising for braiding experiments. Overall, our experiment demonstrates the highly promising nature of the TI Josephson junctions for the Majorana research thanks to the naturally existing spin-momentum locking of the TSS.

## MATERIALS AND METHODS

### Materials and device fabrication

Bulk single crystals of BiSbTeSe_2_, with nominal stoichiometry of Bi:Sb:Te:Se = 1:1:1:2, were synthesized by the modified Bridgman method using high-purity (99.9999%) Bi, Sb, Te, and Se as starting materials by following the recipe described in (*34*). We mechanically exfoliated thin (10 to 20 nm) BiSbTeSe_2_ flakes under atmospheric conditions from bulk single crystals on degenerately doped Si wafers covered by 280-nm SiO_2_. The latter acts as dielectric for the back gate. Before the exfoliation, the substrates were cleaned with acetone, isopropanol, and a low-power ozone treatment. BiSbTeSe_2_ flakes suitable for device fabrication were identified under an optical microscope. The junctions were defined by electron beam lithography. Poly(methyl methacrylate) A4 resist was exposed using a Raith PIONEER Two system. After development, residual resist in the contact areas was removed by a gentle oxygen plasma treatment. Subsequent cleaning in a hydrochloric acid solution shortly before metallization ensured the removal of any oxide layer. Then, 3 nm + 45 nm Ti/Al contacts were thermally evaporated after a gentle in situ Ar cleaning to remove light atmospheric adsorbates, and the device was exposed to air to form a native Al_2_O_3_ layer. Because of the bulk-insulating nature of our BiSbTeSe_2_, the SC proximity effect only affects the surface in direct contact with the superconductor. An additional atomic layer deposition of a thin (≤1 nm) Al_2_O_3_ layer onto the whole device was performed at 80°C using Ultratec Savannah S200 to form a tunnel barrier. For the fabrication of the tunnel electrodes, another electron beam lithography was performed to define the pattern. A 5-nm-thick Pt layer was sputter-deposited to act as wetting layer, followed by sputtering an additional 45-nm-thick Au layer. The contact resistances achieved by this procedure are typically larger than 100 kohms, ensuring electron tunneling to be the prevalent form of transport. While we can reliably produce Josephson junctions, the success rate of the tunnel junctions on top of them is less than 50%, making it challenging to fabricate devices with two working tunnel contacts. The main reason for this is the very small required thickness of our dielectric, making it very sensitive to variations in both directions. The precise device geometry was determined by scanning electron microscopy after the measurements were finished.

### Measurements

Transport measurements were performed at base temperature (∼50 mK) of our dry dilution refrigerator (Oxford Instruments TRITON 300). Electrical lines incorporate RC and copper powder filters to reduce noise. Using a two-probe configuration, the differential conductance d*I*/d*V* was measured between each individual tunnel electrode and the SC electrode with a standard low-frequency lock-in technique using a Basel Instruments current preamplifier. A small dc offset introduced by the preamplifier was numerically corrected for after the measurements. An ac voltage of 5 to 10 μV was superimposed to a dc bias voltage. For investigations of the magnetic field dependence, we used our integrated 6/1/1-T SC vector magnet.

### Simulations

To qualitatively determine the differential conductance of the TI Josephson junction, a transport simulation for a 3D tight-binding experiment was performed using the Python library Kwant (*55*). The simulation of a 3D bulk TI is necessary to circumvent the Nielsen-Ninomiya (fermion-doubling) theorem (*56*). The 3D simulation allows us to realize a single Dirac cone on each face of the TI, with the fermion-doubled partner on the opposite surface, where it can be gapped away by applying magnetic fields or opening an SC gap (*57*). As a model for the 3D strong TI, the Bernevig-Hughes-Zhang (BHZ) model was chosen and implemented on a cubic lattice. Superconductivity was included in the Bogoliubov–de-Gennes (BdG) formalism. The system has been extended infinitely along the junction and probed by a tunnel lead attached perpendicularly to the junction. In the basis $\Psi ={\left[\left(\Psi_{↑}^{1},\Psi_{↓}^{1}),(\Psi_{↑}^{2},\Psi_{↓}^{2}\right)\right]}^{T}$, where *i* = 1, 2 is denoting the orbital and ↑,↓ the spin, the full BHZ Hamiltonian is given by

$$H_{\text{BHZ}}=\left[\epsilon_{0}\left(k\right)-\mu \right]+M\left(k\right)\;{\hat{\pi }}_{z}+A_{1}\;k_{z}\;{\hat{\pi }}_{x}{\hat{\sigma }}_{z}+A_{2}\left(k_{x}{\hat{\pi }}_{x}{\hat{\sigma }}_{x}+k_{y}\;{\hat{\pi }}_{x}{\hat{\sigma }}_{y}\right)$$ (4)

with

$$\epsilon_{0}\left(k\right)=C+D_{1}k_{z}^{2}+D_{2}\left(k_{x}^{2}+k_{y}^{2}\right)\;\text{and}$$ (5)

$$M\left(k\right)=M-B_{1}k_{z}^{2}-B_{2}\left(k_{x}^{2}+k_{y}^{2}\right)$$ (6)

The Pauli operators $\hat{\pi }$ act on the orbital degree of freedom and $\hat{\sigma }$ on the spin degree of freedom. In our notation, tensor products of different Pauli operators are always implied, and identity operators acting are not written explicitly. For our purposes, we use the simplest version of the model in Eq. 4 that exhibits a topological phase. We neglect the spin and orbital independent part of the spectrum ϵ_0_(***k***) by setting *C* = *D_j_* = 0 and choose

$$A_{1}=A_{2}=v\;\text{and}\;B_{1}=B_{2}=\frac{v}{2|M|}$$ (7)

With this choice of parameters, the Hamiltonian simplifies to

$$H_{\text{BHZ}}=-\mu +\left(M-\frac{v}{2|M|}\;k^{2}\right){\hat{\pi }}_{z}+v{\hat{\pi }}_{x}\;k\cdot \hat{\sigma }$$ (8)

The model exhibits a topological gap 2|*M*| and a Dirac surface spectrum with the Dirac velocity *v*, which is set to 1 in the simulation. Furthermore, the parameter *M* was set to 1. The reduced model is implemented as a tight-binding model on a cubic lattice in Kwant. The full simulation of the bulk of the TI is necessary to obtain correct surface states. For the inclusion of superconductivity in the system, the time reversed states of the BHZ model were implemented as additional orbitals to explicitly simulate a BdG Hamiltonian. On the surface of the TI, a finite SC gap Δ = 0.1*e*^*i*φ^ is opened by coupling the two sectors of the BdG-Hamiltonian. Two SC regions are separated by a bare TI surface of fixed width on the top of the sample, thus modeling a Josephson junction of the surfaces by varying φ across the junction. Only on that strip of bare TI surface the gapless surface state are found. On all other surfaces, the surface states are gapped away by the finite superconductivity. The phase variation created by the magnetic field in the experiment is implemented directly. On the bottom surface, the phase jump is split up into two steps to prevent hybridization between the surface state in the TI JJ and unphysical ABS on the lower surface of the sample. The cross-sectional geometry is sketched in fig. S12. The dimensions of the simulation in units of the lattice constant are *L* = 9, *L*_SC_ = 8, $L_{\text{SC}}^{\prime }=1$, *L*_T_ = 9, *D* = 12, and *D*_SC_ = 1. The system is extended infinitely along the junction and probed by a tunnel lead attached vertically to the bare TI region at one point. The tunnel lead is simulated as a normal conducting cubic lattice, and the tunnel contact is tuned by varying the hopping from the lead into the system. The differential conductivity is calculated as a function of the energy *E* compared to the SC gap Δ and the phase difference across the Josephson junction ϕ*_B_*.

## Supplementary Materials

### This PDF file includes:

Supplementary Text Figs. S1 to S13 References

## References

[1] M. Sato, Y. Ando, Topological superconductors: A review. Rep. Prog. Phys. 80, 076501 (2017). 10.1088/1361-6633/aa6ac7 28367833

[2] A. Y. Kitaev, Unpaired Majorana fermions in quantum wires. Phys.-Uspekhi 44, 131–136 (2001).

[3] N. Read, D. Green, Paired states of fermions in two dimensions with breaking of parity and time-reversal symmetries and the fractional quantum Hall effect. Phys. Rev. B 61, 10267–10297 (2000).

[4] D. A. Ivanov, Non-abelian statistics of half-quantum vortices in *p*-wave superconductors. Phys. Rev. Lett. 86, 268–271 (2001). 10.1103/PhysRevLett.86.268 11177808

[5] C. Nayak, S. H. Simon, A. Stern, M. Freedman, S. Das Sarma, Non-Abelian anyons and topological quantum computation. Rev. Mod. Phys. 80, 1083–1159 (2008).

[6] J. Alicea, New directions in the pursuit of Majorana fermions in solid state systems. Rep. Prog. Phys. 75, 076501 (2012). 10.1088/0034-4885/75/7/076501 22790778

[7] K. Flensberg, F. von Oppen, A. Stern, Engineered platforms for topological superconductivity and Majorana zero modes. Nat. Rev. Mater. 6, 944–958 (2021).

[8] L. Fu, C. L. Kane, Superconducting proximity effect and majorana fermions at the surface of a topological insulator. Phys. Rev. Lett. 100, 096407 (2008). 10.1103/PhysRevLett.100.096407 18352737

[9] J. R. Williams, A. J. Bestwick, P. Gallagher, S. S. Hong, Y. Cui, A. S. Bleich, J. G. Analytis, I. R. Fisher, D. Goldhaber-Gordon, Unconventional Josephson effect in hybrid superconductor-topological insulator devices. Phys. Rev. Lett. 109, 056803 (2012). 10.1103/PhysRevLett.109.056803 23006196

[10] C. Kurter, A. D. K. Finck, Y. S. Hor, D. J. van Harlingen, Evidence for an anomalous current-phase relation in topological insulator Josephson junctions. Nat. Commun. 6, 7130 (2015). 10.1038/ncomms8130 26027559

[11] G. Yue, C. Zhang, E. D. Huemiller, J. H. Montone, G. R. Arias, D. G. Wild, J. Y. Zhang, D. R. Hamilton, X. Yuan, X. Yao, D. Jain, J. Moon, M. Salehi, N. Koirala, S. Oh, D. J. Van Harlingen, Signatures of Majorana bound states in the diffraction patterns of extended superconductor–topological insulator–superconductor Josephson junctions. Phys. Rev. B 109, 094511 (2024).

[12] J. Wiedenmann, E. Bocquillon, R. S. Deacon, S. Hartinger, O. Herrmann, T. M. Klapwijk, L. Maier, C. Ames, C. Brune, C. Gould, A. Oiwa, K. Ishibashi, S. Tarucha, H. Buhmann, L. W. Molenkamp, 4π-Periodic Josephson supercurrent in HgTe-based topological Josephson junctions. Nat. Commun. 7, 10303 (2016). 10.1038/ncomms10303 PMC4735757 26792013

[13] R. S. Deacon, J. Wiedenmann, E. Bocquillon, F. Dominguez, T. M. Klapwijk, P. Leubner, C. Brune, E. M. Hankiewicz, S. Tarucha, K. Ishibashi, H. Buhmann, L. W. Molenkamp, Josephson radiation from gapless andreev bound states in HgTe-based topological junctions. Phys. Rev. X 7, 021011 (2017).

[14] P. Schüffelgen, D. Rosenbach, C. Li, T. W. Schmitt, M. Schleenvoigt, A. R. Jalil, S. Schmitt, J. Kölzer, M. Wang, B. Bennemann, U. Parlak, L. Kibkalo, S. Trellenkamp, T. Grap, D. Meertens, M. Luysberg, G. Mussler, E. Berenschot, N. Tas, A. A. Golubov, A. Brinkman, T. Schäpers, D. Grützmacher, Selective area growth and stencil lithography for in situ fabricated quantum devices. Nat. Nanotechnol. 14, 825–831 (2019). 10.1038/s41565-019-0506-y 31358942

[15] J.-P. Xu, M.-X. Wang, Z. L. Liu, J.-F. Ge, X. Yang, C. Liu, Z. A. Xu, D. Guan, C. L. Gao, D. Qian, Y. Liu, Q.-H. Wang, F.-C. Zhang, Q.-K. Xue, J.-F. Jia, Experimental detection of a majorana mode in the core of a magnetic vortex inside a topological insulator-superconductor Bi_2_Te_3_/NbSe_2_ heterostructure. Phys. Rev. Lett. 114, 017001 (2015). 10.1103/PhysRevLett.114.017001 25615497

[16] P. Krogstrup, N. L. B. Ziino, W. Chang, S. M. Albrecht, M. H. Madsen, E. Johnson, J. Nygård, C. M. Marcus, T. S. Jespersen, Epitaxy of semiconductor-superconductor nanowires. Nat. Mater. 14, 400–406 (2014). 10.1038/nmat4176 25581626

[17] M. Aghaee, A. Akkala, Z. Alam, R. Ali, A. Alcaraz Ramirez, M. Andrzejczuk, A. E. Antipov, P. Aseev, M. Astafev, B. Bauer, J. Becker, S. Boddapati, F. Boekhout, J. Bommer, T. Bosma, L. Bourdet, S. Boutin, P. Caroff, L. Casparis, M. Cassidy, S. Chatoor, A. W. Christensen, N. Clay, W. S. Cole, F. Corsetti, A. Cui, P. Dalampiras, A. Dokania, G. de Lange, M. de Moor, J. C. Estrada Saldaña, S. Fallahi, Z. H. Fathabad, J. Gamble, G. Gardner, D. Govender, F. Griggio, R. Grigoryan, S. Gronin, J. Gukelberger, E. B. Hansen, S. Heedt, J. Herranz Zamorano, S. Ho, U. L. Holgaard, H. Ingerslev, L. Johansson, J. Jones, R. Kallaher, F. Karimi, T. Karzig, C. King, M. E. Kloster, C. Knapp, D. Kocon, J. Koski, P. Kostamo, P. Krogstrup, M. Kumar, T. Laeven, T. Larsen, K. Li, T. Lindemann, J. Love, R. Lutchyn, M. H. Madsen, M. Manfra, S. Markussen, E. Martinez, R. McNeil, E. Memisevic, T. Morgan, A. Mullally, C. Nayak, J. Nielsen, W. H. P. Nielsen, B. Nijholt, A. Nurmohamed, E. O’Farrell, K. Otani, S. Pauka, K. Petersson, L. Petit, D. I. Pikulin, F. Preiss, M. Quintero-Perez, M. Rajpalke, K. Rasmussen, D. Razmadze, O. Reentila, D. Reilly, R. Rouse, I. Sadovskyy, L. Sainiemi, S. Schreppler, V. Sidorkin, A. Singh, S. Singh, S. Sinha, P. Sohr, T. Stankevič, L. Stek, H. Suominen, J. Suter, V. Svidenko, S. Teicher, M. Temuerhan, N. Thiyagarajah, R. Tholapi, M. Thomas, E. Toomey, S. Upadhyay, I. Urban, S. Vaitiekėnas, K. Van Hoogdalem, D. V. Viazmitinov, S. Waddy, D. Van Woerkom, D. Vogel, J. Watson, J. Weston, G. W. Winkler, C. K. Yang, S. Yau, D. Yi, E. Yucelen, A. Webster, R. Zeisel, R. Zhao, InAs-Al hybrid devices passing the topological gap protocol. Phys. Rev. B 107, 245423 (2023).

[18] M. Valentini, F. Peñaranda, A. Hofmann, M. Brauns, R. Hauschild, P. Krogstrup, P. San-Jose, E. Prada, R. Aguado, G. Katsaros, Nontopological zero-bias peaks in full-shell nanowires induced by flux-tunable Andreev states. Science 373, 82–88 (2021). 10.1126/science.abf1513 34210881

[19] S. Das Sarma, In search of Majorana. Nat. Phys. 19, 165–170 (2023).

[20] R. Hess, H. F. Legg, D. Loss, J. Klinovaja, Trivial andreev band mimicking topological bulk gap reopening in the nonlocal conductance of long rashba nanowires. Phys. Rev. Lett. 130, 207001 (2023). 10.1103/PhysRevLett.130.207001 37267549

[21] A. R. Akhmerov, J. P. Dahlhaus, F. Hassler, M. Wimmer, C. W. J. Beenakker, Quantized conductance at the Majorana phase transition in a disordered superconducting wire. Phys. Rev. Lett. 106, 057001 (2011). 10.1103/PhysRevLett.106.057001 21405421

[22] T. O. Rosdahl, A. Vuik, M. Kjaergaard, A. R. Akhmerov, Andreev rectifier: A nonlocal conductance signature of topological phase transitions. Phys. Rev. B 97, 045421 (2018).

[23] D. I. Pikulin, B. van Heck, T. Karzig, E. A. Martinez, B. Nijholt, T. Laeven, G. W. Winkler, J. D. Watson, S. Heedt, M. Temurhan, V. Svidenko, R. M. Lutchyn, M. Thomas, G. de Lange, L. Casparis, C. Nayak, Protocol to identify a topological superconducting phase in a three-terminal device. arXiv:2103.12217 [cond-mat.mes-hall] (2021).

[24] A. Banerjee, O. Lesser, M. A. Rahman, C. Thomas, T. Wang, M. J. Manfra, E. Berg, Y. Oreg, A. Stern, C. M. Marcus, Local and nonlocal transport spectroscopy in planar josephson junctions. Phys. Rev. Lett. 130, 096202 (2023). 10.1103/PhysRevLett.130.096202 36930915

[25] A. Banerjee, O. Lesser, M. A. Rahman, H.-R. Wang, M.-R. Li, A. Kringhøj, A. M. Whiticar, A. C. C. Drachmann, C. Thomas, T. Wang, M. J. Manfra, E. Berg, Y. Oreg, A. Stern, C. M. Marcus, Signatures of a topological phase transition in a planar Josephson junction. Phys. Rev. B 107, 245304 (2023).

[26] G. Tkachov, E. M. Hankiewicz, Helical Andreev bound states and superconducting Klein tunneling in topological insulator Josephson junctions. Phys. Rev. B 88, 075401 (2013).

[27] A. C. Potter, L. Fu, Anomalous supercurrent from Majorana states in topological insulator Josephson junctions. Phys. Rev. B 88, 121109 (2013).

[28] H. le Sueur, P. Joyez, H. Pothier, C. Urbina, D. Esteve, Phase controlled superconducting proximity effect probed by tunneling spectroscopy. Phys. Rev. Lett. 100, 197002 (2008). 10.1103/PhysRevLett.100.197002 18518479

[29] J.-D. Pillet, C. H. Quay, P. Morfin, C. Bena, A. L. Yeyati, P. Joyez, Andreev bound states in supercurrent-carrying carbon nanotubes revealed. Nat. Phys. 6, 965–969 (2010).

[30] L. Bretheau, J. I.-J. Wang, R. Pisoni, K. Watanabe, T. Taniguchi, P. Jarillo-Herrero, Tunnelling spectroscopy of Andreev states in graphene. Nat. Phys. 13, 756–760 (2017).

[31] H. Ren, F. Pientka, S. Hart, A. T. Pierce, M. Kosowsky, L. Lunczer, R. Schlereth, B. Scharf, E. M. Hankiewicz, L. W. Molenkamp, B. I. Halperin, A. Yacoby, Topological superconductivity in a phase-controlled Josephson junction. Nature 569, 93–98 (2019). 10.1038/s41586-019-1148-9 31019296

[32] A. Fornieri, A. M. Whiticar, F. Setiawan, E. P. Marín, A. C. C. Drachmann, A. Keselman, S. Gronin, C. Thomas, T. Wang, R. L. Kallaher, G. C. Gardner, E. Berg, E. Berg, M. J. Manfra, A. Stern, C. M. Marcus, F. Nichele, Evidence of topological superconductivity in planar Josephson junctions. Nature 569, 89–92 (2019). 10.1038/s41586-019-1068-8 31019303

[33] F. Nichele, E. Portolés, A. Fornieri, A. M. Whiticar, A. C. C. Drachmann, S. Gronin, T. Wang, G. C. Gardner, C. Thomas, A. T. Hatke, M. J. Manfra, C. M. Marcus, Relating Andreev bound states and supercurrents in hybrid Josephson junctions. Phys. Rev. Lett. 124, 226801 (2020). 10.1103/PhysRevLett.124.226801 32567899

[34] Z. Ren, A. A. Taskin, S. Sasaki, K. Segawa, Y. Ando, Optimizing Bi_2−*x*_SB*_x_*Te_3−*y*_Se*_y_* solid solutions to approach the intrinsic topological insulator regime. Phys. Rev. B 84, 165311 (2011).

[35] T. Arakane, T. Sato, S. Souma, K. Kosaka, K. Nakayama, M. Komatsu, T. Takahashi, Z. Ren, K. Segawa, Y. Ando, Tunable Dirac cone in the topological insulator Bi_2−*x*_Sb*_x_*Te_3−*y*_Se*_y_*. Nat. Commun. 3, 636 (2012). 10.1038/ncomms1639 22273674

[36] S. Ghatak, O. Breunig, F. Yang, Z. Wang, A. A. Taskin, Y. Ando, Anomalous Fraunhofer patterns in gated Josephson junctions based on the bulk-insulating topological insulator BiSbTeSe_2_. Nano Lett. 18, 5124–5131 (2018). 10.1021/acs.nanolett.8b02029 30028140

[37] C. W. J. Beenakker, Universal limit of critical-current fluctuations in mesoscopic Josephson junctions. Phys. Rev. Lett. 67, 3836–3839 (1991). 10.1103/PhysRevLett.67.3836 10044838

[38] S. Pilgram, W. Belzig, C. Bruder, Excitation spectrum of mesoscopic proximity structures. Phys. Rev. B 62, 12462–12467 (2000).

[39] T. Laeven, B. Nijholt, M. Wimmer, A. R. Akhmerov, Enhanced proximity effect in zigzag-shaped Majorana Josephson junctions. Phys. Rev. Lett. 125, 086802 (2020). 10.1103/PhysRevLett.125.086802 32909794

[40] I. Martin, D. Mozyrsky, Nonequilibrium theory of tunneling into a localized state in a superconductor. Phys. Rev. B 90, 100508 (2014).

[41] J. Stenger, T. D. Stanescu, Tunneling conductance in semiconductor-superconductor hybrid structures. Phys. Rev. B 96, 214516 (2017).

[42] A. Melo, C.-X. Liu, P. Rożek, T. Örn Rosdahl, M. Wimmer, Conductance asymmetries in mesoscopic superconducting devices due to finite bias. SciPost Phys. 10, 037 (2021).

[43] C. M. Moehle, P. K. Rout, N. A. Jainandunsing, D. Kuiri, C. T. Ke, D. Xiao, C. Thomas, M. J. Manfra, M. P. Nowak, S. Goswami, Controlling Andreev bound states with the magnetic vector potential. Nano Lett. 22, 8601–8607 (2022). 10.1021/acs.nanolett.2c03130 PMC9650727 36279222

[44] A. Banerjee, M. Geier, M. A. Rahman, D. S. Sanchez, C. Thomas, T. Wang, M. J. Manfra, K. Flensberg, C. M. Marcus, Control of Andreev bound states using superconducting phase texture. Phys. Rev. Lett. 130, 116203 (2023). 10.1103/PhysRevLett.130.116203 37001098

[45] D. O. Oriekhov, Y. Cheipesh, C. W. J. Beenakker, Voltage staircase in a current-biased quantum-dot Josephson junction. Phys. Rev. B 103, 094518 (2021).

[46] F. Zhou, P. Charlat, B. Spivak, B. Pannetier, Density of states in superconductor-normal metal-superconductor junctions. J. Low Temp. Phys. 110, 841–850 (1998).

[47] C. W. J. Beenakker, Random-matrix theory of quantum transport. Rev. Mod. Phys. 69, 731–808 (1997).

[48] A. A. Taskin, Z. Ren, S. Sasaki, K. Segawa, Y. Ando, Observation of dirac holes and electrons in a topological insulator. Phys. Rev. Lett. 107, 016801 (2011). 10.1103/PhysRevLett.107.016801 21797561

[49] H. Kim, S.-J. Choi, H.-S. Sim, S. Park, Boundary-induced Majorana coupling in a planar topological Josephson junction. Phys. Rev. B 111, 045414 (2025).

[50] E. Nikodem, J. Schluck, H. F. Legg, M. Geier, M. Papaj, M. Bagchi, L. Fu, Y. Ando, Topological insulator nano-SQUID: Flux-tunable platform for topological superconductivity. arXiv:2412.07993 [cond-mat.mes-hall] (2025).

[51] A. Murani, B. Dassonneville, A. Kasumov, J. Basset, M. Ferrier, R. Deblock, S. Guéron, H. Bouchiat, Microwave signature of topological Andreev level crossings in a bismuth-based Josephson junction. Phys. Rev. Lett. 122, 076802 (2019). 10.1103/PhysRevLett.122.076802 30848609

[52] G. Ben-Shach, A. Haim, I. Appelbaum, Y. Oreg, A. Yacoby, B. I. Halperin, Detecting Majorana modes in one-dimensional wires by charge sensing. Phys. Rev. B 91, 045403 (2015).

[53] S. S. Hegde, G. Yue, Y. Wang, E. Huemiller, D. J. Van Harlingen, S. Vishveshwara, A topological Josephson junction platform for creating, manipulating, and braiding Majorana bound states. Ann. Phys. 423, 168326 (2020).

[54] E. Grosfeld, A. Stern, Observing Majorana bound states of Josephson vortices in topological superconductors. Proc. Natl. Acad. Sci. U.S.A. 108, 11810–11814 (2011). 10.1073/pnas.1101469108 PMC3141995 21730165

[55] C. W. Groth, M. Wimmer, A. R. Akhmerov, X. Waintal, Kwant: A software package for quantum transport. New J. Phys. 16, 063065 (2014).

[56] H. B. Nielsen, M. Ninomiya, The Adler-Bell-Jackiw anomaly and Weyl fermions in a crystal. Phys. Lett. B 130, 389–396 (1983).

[57] A. Ziesen, I. C. Fulga, F. Hassler, Geometry-independent tight-binding method for massless Dirac fermions in two dimensions. Phys. Rev. B 107, 195409 (2023).

[58] R. C. Dynes, V. Narayanamurti, J. P. Garno, Direct measurement of quasiparticle-lifetime broadening in a strong-coupled superconductor. Phys. Rev. Lett. 41, 1509–1512 (1978).

[59] W. Zhang, R. Yu, H.-J. Zhang, X. Dai, Z. Fang, First-principles studies of the three-dimensional strong topological insulators Bi−2Te_3_, Bi_2_*Se*_3_ and Sb_2_Te_3_. New J. Phys. 12, 065013 (2010).

[60] D. López-Núñez, A. Torras-Coloma, Q. Portell-Montserrat, E. Bertoldo, L. Cozzolino, G. A. Ummarino, A. Zaccone, G. Rius, M. Martínez, P. Forn-Díaz, Superconducting penetration depth of aluminum thin films. Supercond. Sci. Technol. 38, 095004 (2025).

[61] L. F. Shatz, C. W. Christensen, Numerical inductance calculations based on first principles. PLOS ONE 9, e111643 (2014). 10.1371/journal.pone.0111643 PMC4234313 25402467

[62] J. Brede, M. Bagchi, A. Greichgauer, A. Uday, A. Bliesener, G. Lippertz, R. Yazdanpanah, A. Taskin, Y. Ando, Characterizing the chemical potential disorder in the topological insulator (Bi_1−*x*_SB*_x_*)_2_Te_3_ thin films. Phys. Rev. Mater. 8, 104202 (2024).

[63] A. Montag, Master’s thesis, RWTH Aachen University (2023); 10.5281/zenodo.11615512.

