# Supplementary material for: Tunneling spectroscopy of topological insulator Josephson junctions
Source: Sci Adv. 2026 Oct 30;12(44):eaeg0687. doi: 10.1126/sciadv.aeg0687 (PMC13721961; doi:10.1126/sciadv.aeg0687)
Supplement: Supplementary file 1 [file sciadv.aeg0687_sm.pdf]

Science Advances
AAAS

Supplementary Materials for

Tunneling spectroscopy of topological insulator Josephson junctions

Jakob Schluck *et al.*

Corresponding author: Jakob Schluck, schluck@ph2.uni-koeln.de; Yoichi Ando, ando@ph2.uni-koeln.de

Sci. Adv. **12**, eaeg0687 (2026)
DOI: 10.1126/sciadv.aeg0687

This PDF file includes:

Supplementary Text
Figs. S1 to S13
References

Supplementary Text

S1 Critical temperature of the superconducting film

In Fig. S1, we show a four-point resistance measurement of a pure aluminum film, which was deposited under the same conditions as the one used for the device fabrication. We see a sharp drop in resistance at $T_c \approx 1.15K$, indicating the superconducting phase transition. Via the standard BCS formula $\Delta = 1.76k_BT_C$, this gives $\Delta \approx 170\,\mu$eV for the aluminum film.

S2 Tunneling data of pure aluminum film

In Fig. S2, we show data from a different device, where the tunnel contact was not in contact with a topological insulator, but only probing the superconducting film itself. The resulting experimental curve is well described using the typical Dynes equation for the density of states N_s (*58*) and calculating the tunneling current by convolving it with the derivative of the Fermi-Dirac distribution at the given temperature.

$$N_s(E) \propto \left| \Re \left(\frac{E - i\Gamma}{\sqrt{(E - i\Gamma)^2 - \Delta^2}} \right) \right| \tag{S1}$$

$$\frac{dI}{dV}(V) \propto \int_{-\infty}^{\infty} N_s(E) \left(-\frac{\partial f(E - eV)}{\partial E} \right) dE \tag{S2}$$

We find good agreement for $T_c = 1.05\,\mathrm{K}$, $T = 0.05\,\mathrm{K}$, and $\Gamma = 8.8\,\mu$eV. For comparison, the zero-field data of the tunneling conductance from our topological insulator device is also shown.

S3 Sample geometry and flux focusing

The inner area of the loop is given by $W_L \times H_L = 2.15 \times 3\ \mu\mathrm{m}^2$, while the junction has a width of 3 µm and a length of about 50 nm. The superconducting (SC) electrodes have a width of 400 nm. Making a simple assumption that the flux expulsion from the SC electrodes leads to an increase of the magnetic fluxes for which half the electrode width should be taken into account (*36*), we obtain an expected periodicity of $\Delta B_1 = 196\ \mu$T for TJ1 and $\Delta B_2 = 225\ \mu$T for TJ2, in excellent agreement with our experimental data.

S4 Data in higher magnetic fields

In Fig. S3, we show the $\mathrm{d}I/\mathrm{d}V$ spectra on TJ1 and TJ2 from 0 T to 15 mT, which was the maximum available magnetic field in this experiment due to the limitation of the high-resolution current source (Keithley 2450) used for energizing the superconducting magnet in the very low magnetic-field range. The superconductivity in the Al electrode was strongly suppressed in 15 mT, which can be seen in the suppression of the induced gap. Nevertheless, the dispersion of modes with $\tau > 0.95$ can be observed in the whole magnetic-field range. For our Al films of thickness 45 nm, we typically observe a critical field of ~20 mT. In the right panels we show line cuts at 0 T and at the fields corresponding to $\varphi \approx \pi$ near 1, 2, 3, 4, 5, 6, and 7 mT to demonstrate the robustness with respect to magnetic fields.

S5 Data for higher bias voltages

In Fig. S4, we show the $\mathrm{d}I/\mathrm{d}V$ spectra on TJ1 and TJ2 for the biases voltage V_b up to 1 mV for the entire range of V_G. The general reduction of $\mathrm{d}I/\mathrm{d}V$ at more negative V_G values, reflecting the n-type nature of the TI flake used here, is clearly visible. Also, one can see a sequence of broad impurity resonances occasionally crossing the gap energy for both devices without introducing in-gap states. In both cases, it requires $\Delta V_\mathrm{G} \approx 1 - 2\,\mathrm{V}$ to change their energy by $2\,\mathrm{meV}$, resulting in the gate efficiency given in the main text.

S6 Josephson penetration length

The RF-SQUID geometry of our device does not allow for a direct measurement of its critical current. However, in our previous study of similar devices (*36*), we typically find a critical current per unit width of the Josephson junction $I_c/W \approx 100\,\mathrm{nA/\mu m}$. We use this value to estimate the Josephson penetration length in our device using $\lambda_J = \sqrt{\frac{\Phi_0 W^2 t}{4\pi\mu_0 I_c \lambda^2}}$, where $W = 3\,\mathrm{\mu m}$ is the width of the junction, $t = 2\,\mathrm{nm}$ is the extension of the surface state (*59*), and $\lambda \approx 100\,\mathrm{nm}$ is the London penetration depth for an aluminum film of ~50-nm thickness (*60*). We obtain $\lambda_J \approx 28\,\mathrm{\mu m}$, approximately one order of magnitude larger than our junction width, justifying the assumption of

the linear phase gradient in the junction for finite magnetic fields.

S7 Loop inductance

The geometrical inductance of our RF-SQUID can be estimated using the model cited e.g. in Ref. (*61*). It yields $L_{geo} \approx 6\,\text{pH}$ for the dimensions of our devices. The kinetic inductance of Al films of comparable fabrication and thickness was determined in Ref. (*60*). Using their values, we estimate $L_{kin} \approx 9\,\text{pH}$ for our device. Even if we assume a large critical current of $I_c \approx 1\,\mu\text{A}$, the ratio $(L_{geo} + L_{kin})I_c/\Phi_0 \approx 0.008 << 1$ indicates that the loop inductance will have a marginal impact.

S8 Surface state coherence length

The Fu-Kane theory (*8*) assumed that the Josephson junction is in the short-junction limit. This requires the superconducting coherence length ξ_{TI} in the TI surface state to be larger than the junction length $L = 50\,\text{nm}$. The effective coherence length in a non-ballistic junction can be calculated via $\xi_{\text{TI}} = \sqrt{\frac{\hbar v_F l_e}{2\Delta}}$, with l_e the mean free path in the TI surface. If we take $l_e \approx 40$ nm and $v_F = 4.6 \times 10^5$ m/s from Ref. 48, we obtain $\xi_{\text{TI}} \approx 200$ nm, which is longer than the junction length. Even if we take a much shorter $l_e \approx 5\,\text{nm}$ combined with $v_F = 5.5 \times 10^5$ m/s as in Ref. 36, we obtain $\xi_{\text{TI}} \approx 75\,\text{nm}$, which is still longer than the junction length. Therefore, our junctions are safely in the short-junction limit.

S9 Temperature dependence of the spectra

In Fig. S5, we show the temperature dependence of the spectra for TJ1 and TJ2 at $V_G = 0$. The spectra are essentially robust against increasing temperature, although thermal smearing is evident. Even at $T = 600\,\text{mK}$, the key features are still recognizable.

S10 Confirmation of reproducibility

In Fig. S6, we show the data for TJ2 on the device discussed in the main text (device 1) at $V_G = 0$, as well as the data obtained from another device with an identical design (device 2). It is fabricated on a $BiSbTeSe_2$ flake exfoliated from the same crystal and has tunnel junctions TJ1' and TJ2' in analogous positions. We observe the same closing and reopening of the gap as a function of φ as for device 1.

Similarly, the locality of the spectra is also reproduced as shown in Fig. S7. The periodicity for TJ1' is larger than that for TJ2', which is understood from the geometry as discussed in the main text.

In Fig. S8, the gate stability of the data from device 2 is shown. For TJ1', the phenomenology is the same as discussed for device 1. We observe an apparent gap closing at $\varphi = (2n + 1)\pi$, which in some cases (e.g. $V_G = 5$ V) is accompanied by a broad ZBCP. For TJ2', the situation is more complex. Its conductance becomes strongly suppressed for negative gate voltages, and the strongly asymmetric signals for more positive gate voltages suggests a highly disordered dielectric for this tunnel contact. Nevertheless, the apparent gap closing itself is reproduced.

S11 Additional device with extended gate range

The risk of electrostatic discharge as well as potentially leaky gate dielectrics limited the gating range of the device discussed in the main text. In an additional device fabricated in the same way, we were able to tune the gate voltage over a wider range. In Fig. S9a, we show the normalized differential conductance for both tunnel junctions on this device for $-200\,V \leq V_G \leq 60\,V$. For more negative gate voltages, the gate dielectric started to develop notable leakage current that impeded further measurement. We observe a decrease of conductance by a factor of 2 and 4 at the two tunnel electrodes, larger than the one presented in the main text. However, contrary to the situation in Ref. (*36*), a global minimum, indicating the Dirac point, is not found. We tentatively attribute this to the reduced gating efficiency due to the screening from the metallic tunneling electrode.

To obtain more quantitative information on the position of the chemical potential with respect to the bulk band structure, we performed extended bias sweeps, shown in Figs. S9b,c. The bias

range of ±200 mV studied here should well cover the bulk band gap of $BSTS_2$, which was shown to be about 300 meV large (*35*). The overall gate-tunability is well visible. In particular, the sudden increase in conductance at 90 meV for V_G =0 V clearly shifts towards higher energies with more negative gate voltages. One can reasonably assign this feature to the bottom of the conduction band (*62*). At V_G =-200 V, this feature appears at V_b =150 mV, indicating a shift in the chemical potential of $\Delta\mu$ =60 meV in the range of ΔV_G =200 V. From this we can infer μ <50 meV, following the results of Ref. (*35*). A clearer observation of the bulk band structure is probably impeded by the non-ideal tunnel contact as well as the presence of the Al-electrodes nearby.
Reassuringly, the appearance of strongly dispersing modes, potentially indicating a gap closing and reopening phenomenology, shown in Fig. S9D,E, remains robust also over the entire range of chemical potential studied in this device.

S12 Observation of spurious resonant states

In a device of similar geometry, we observed an induced SC gap, but the spectrum did not change with the phase bias at $V_G = 0$. However, when we swept V_G without applying any phase bias, a sharp and strongly-dispersing Andreev bound state (ABS) appeared in some V_G intervals. An example is shown in Fig. S10a for the V_G-interval of 22.5 – 25 V. The phase-bias dependence of this ABS changes strongly with V_G (Fig. S10b), and interestingly, the state becomes gapless at V_G = 23.6 V where it produced a sharp ZBCP. The electron-hole asymmetry of the ABS switched across V_G = 23.6 V. This behavior is consistent with the model for a quantum-dot state in a Josephson junction being tuned in and out of resonance with the superconductor (*45*). It is useful to note that this ZBCP appears only at particular V_G values and is not accompanied by low-energy excited states, which is completely different from the gap closing phenomenology expected in the Fu-Kane model. As for the origin of this peculiar feature observed in this device, we speculate that due to the uncontrollable nature of our tunnel contacts, the tunnel electrode spuriously did not couple to the TI surface, but rather coupled to a single impurity level in the dielectric with a charging energy that was tuned by V_G.

We can make use of this spurious feature to estimate the energy resolution. In Fig. S11 we present a single line cut at resonance V_G = 23.6 V, close to $\varphi = \pi$ but slightly off. In this plot, the

two ABSs are individually resolved and the peaks appear at ±30 μeV. We therefore conclude that our energy resolution is of the order of 30 μeV, which is much larger than the thermal energy of the measurement temperature (~50 mK) and we therefore attribute it to electronic noise in the setup.

S13 Calculation of the Majorana velocity

The Majorana velocity for the Josephson junction in the short-junction limit depends on the length of the junction. Starting from the surface Hamiltonian of the TI and including superconductivity in the Bogoliubov-de Gennes formalism, the full Hamiltonian can be split into

$$H = \underbrace{H_\perp + H_\pi}_{H_{\mathrm{JR}}} + H_\| \,; \tag{S3}$$

$H_\perp$ and $H_\|$ denote the momentum component orthogonal and parallel to the junction, respectively, and H_π accounts for the superconductivity for a phase difference of $\varphi = \pi$ across the junction. $H_{\mathrm{JR}} = H_\perp + H_\pi$ is a four-band generalization of the Jackiw-Rebbi model and can be solved exactly for two zero-energy Majorana bound states in the junction. The remaining contributions are included perturbatively and by evaluating $H_\|$ to first order we find the Majorana velocity

$$v_M = v_F \frac{\Delta^2}{\Delta^2 + \mu^2} \frac{\cos\left(\frac{\mu L}{\hbar v_F}\right) + \frac{\Delta}{\mu} \sin\left(\frac{\mu L}{\hbar v_F}\right)}{1 + \frac{\Delta L}{\hbar v_F}} \tag{S4}$$

with the TI surface velocity v_F, the magnitude of the superconducting gap Δ, the chemical potential μ, and the length of the junction L. For more information, see Eq. (3.23) of Ref. 63. In the limit $L \ll \hbar v_F/\Delta$, it reduces to the result presented by Fu and Kane in Ref. 8. Moreover, for $\mu \gg \Delta$, we obtain the simple expression $v_M = v_F(\Delta/\mu)^2$ mentioned in Ref. 27. We note that the assumption of a single transversal mode, which lies behind the calculations, is valid for $\mu < \hbar v_F/L$.

S14 Theoretical simulation at fixed phase

The theoretical simulations presented in the main text were performed by taking a running average over the phase in a small window of $\delta\varphi \approx 0.15$. This approach was adopted to capture the phase uncertainty (either along the junction or in time) of the experimental situation. The peak at zero

bias seen in Fig. 2(b) for $\varphi = \pi$ in the main text is not a signature of a zero mode. Rather, it is a broadened version of a BCS peak, which appears for any slight deviation from the exact value of $\varphi = \pi$.

To demonstrate this point, we show in Fig. S13 the result of the same simulation as that for Fig. 2(b), but without applying a running average over the phase. Note that the curve for $\varphi = 0$ (blue) remains essentially unchanged, whereas the zero-bias peak for $\varphi = \pi$ disappears in Fig. S13 and is replaced by a nearly flat dependence (red). This calculation clearly shows that the zero-bias feature at $\varphi = \pi$ is highly sensitive to experimental conditions, such as whether there is an exact phase bias or some averaging over a small phase window. Importantly, this sensitivity of the spectrum to a small phase variation occurs only near $\varphi = \pi$, while the general features of the gap closing discussed in the main text remain robust.

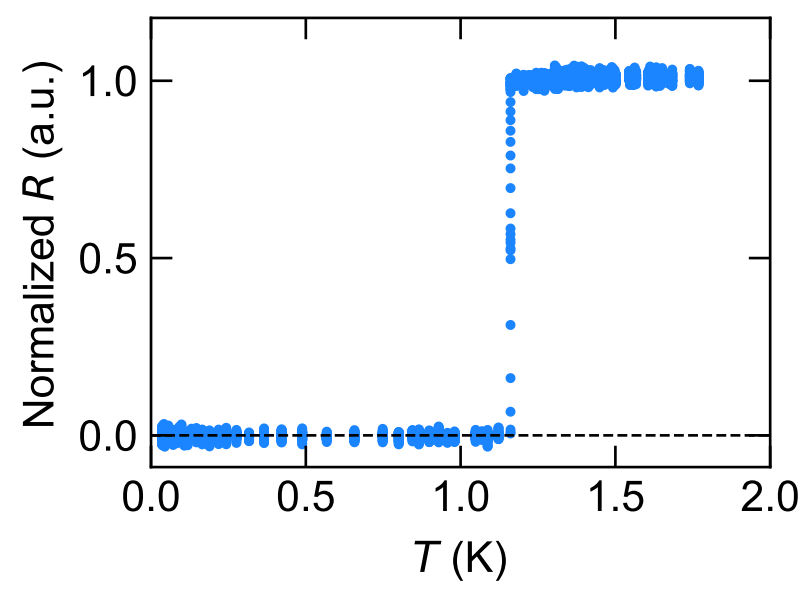

Figure S1: Temperature dependence of the resistance of the Al film measured in a four-probe geometry. The critical temperature is found to be ≈ 1.1K.

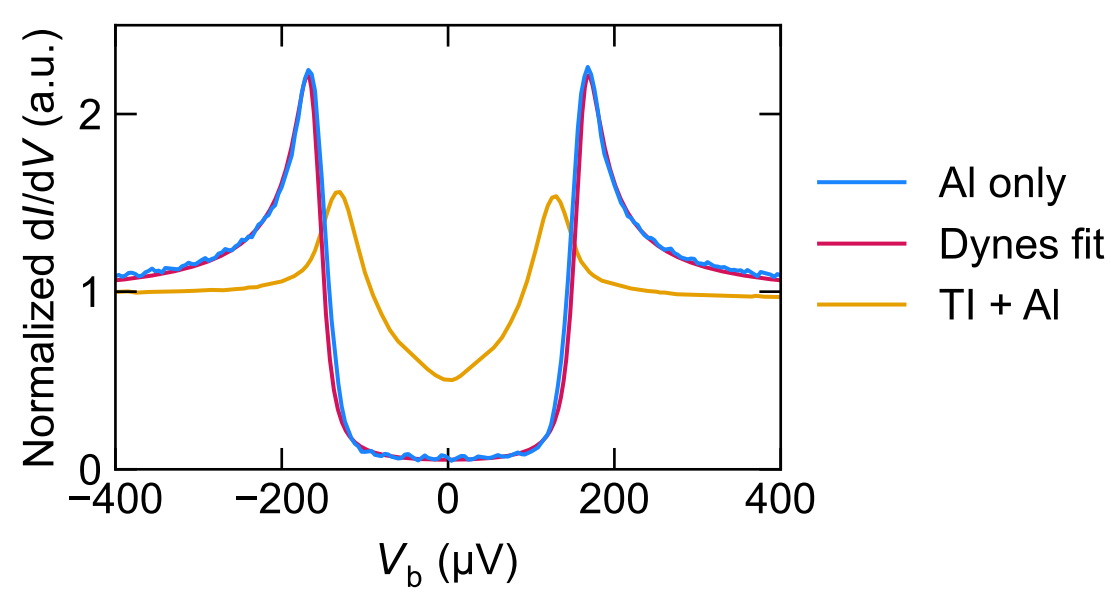

Figure S2: $\mathrm{d}I/\mathrm{d}V$ spectra measured at a different device with no TI coupled to the aluminum (blue curve) in comparison to the device studied here (yellow curve). The Al only data is well described by a standard Dynes model using T_c = 1.05 K, T = 0.05 K, and Γ = 8.8 μeV

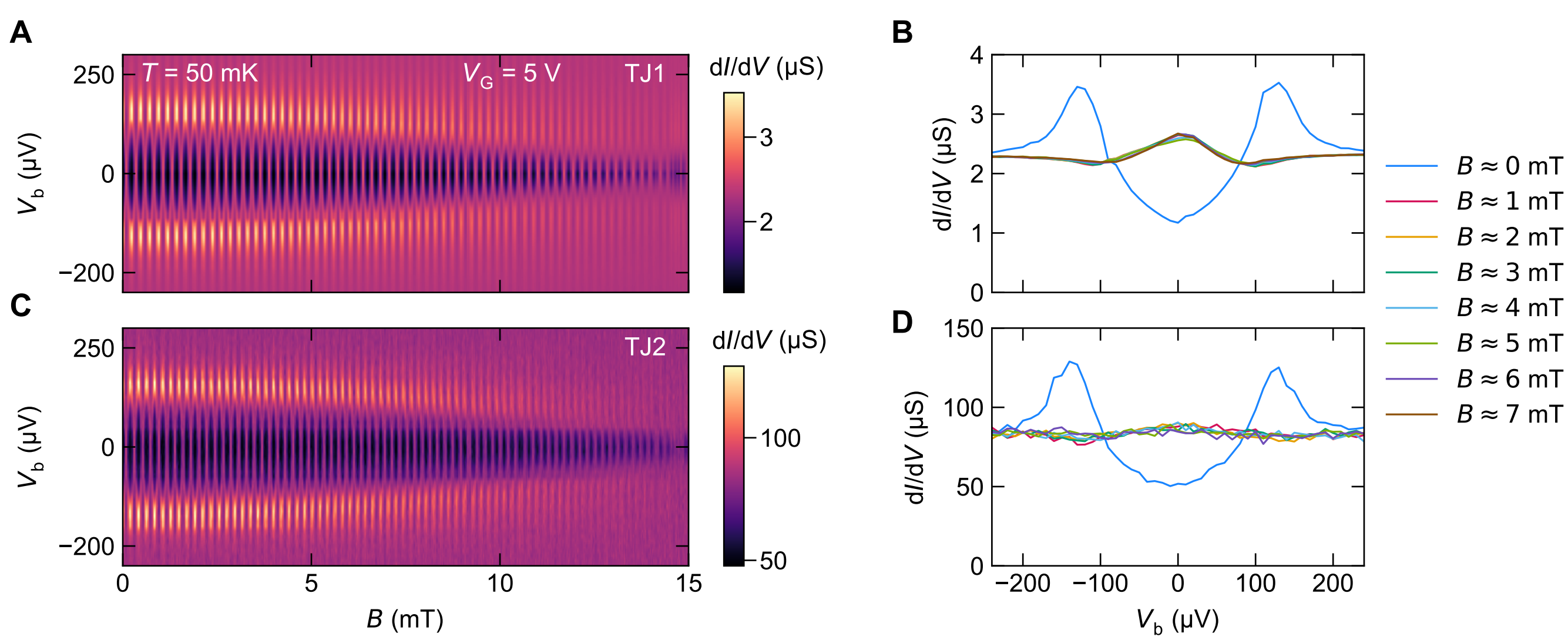

Figure S3: **A,C,** $\mathrm{d}I/\mathrm{d}V$ spectra measured at TJ1 and TJ2 of the device discussed in the main text (device 1) for the full magnetic-field range that was available in this experiment. A gradual reduction of the induced gap is observed in both junctions. **B,D,** Line cuts of the data in panels **A** and **B** at 0 T and at the fields near 1, 2, 3, 4, 5, 6, and 7 mT corresponding to $\varphi \approx \pi$.

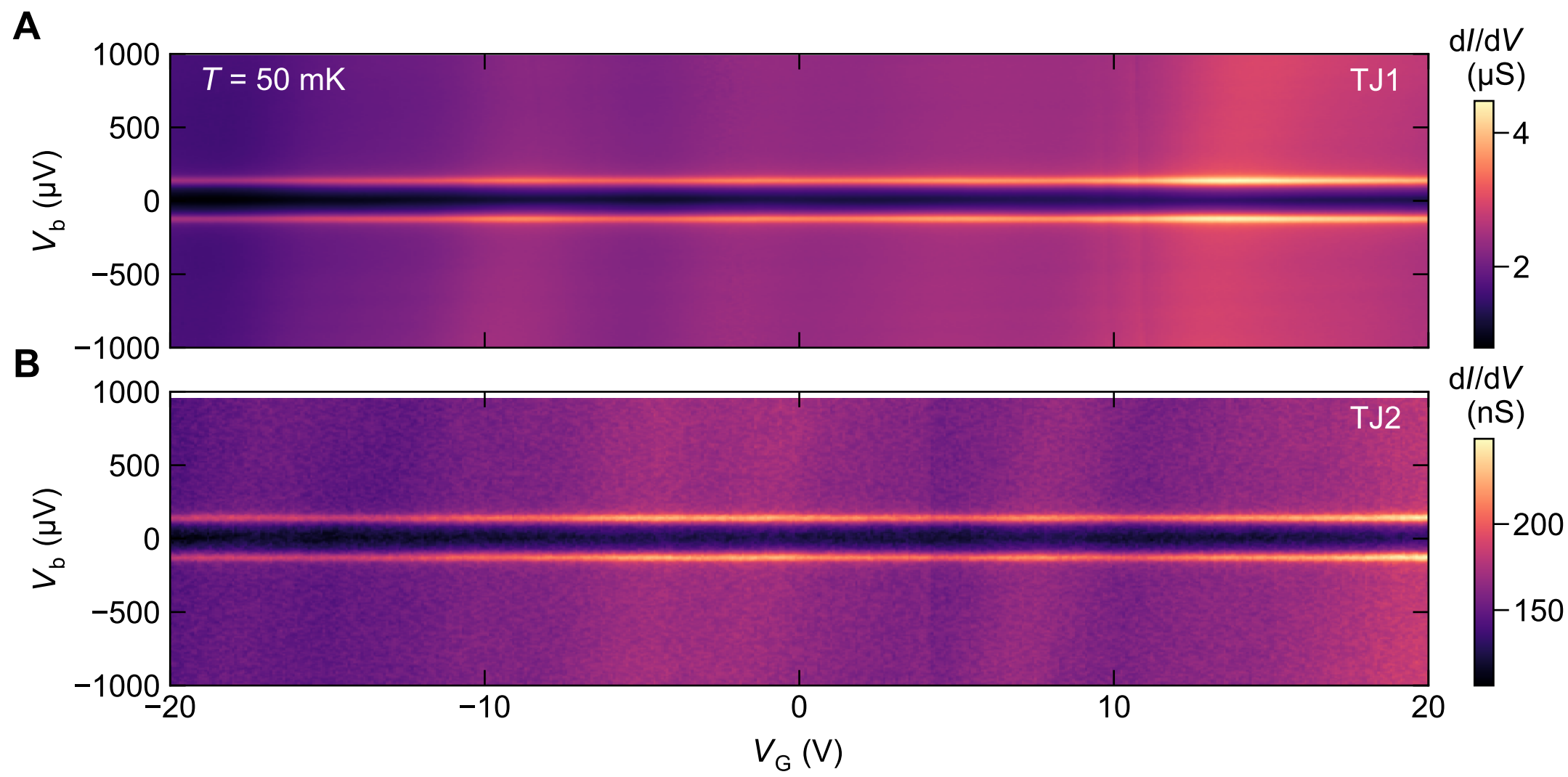

Figure S4: $\mathrm{d}I/\mathrm{d}V$ spectra measured at TJ1 (**A**) and TJ2 (**B**) of the device discussed in the main text (device 1) for a larger bias-voltage range (±1 mV) and without normalizing the data.

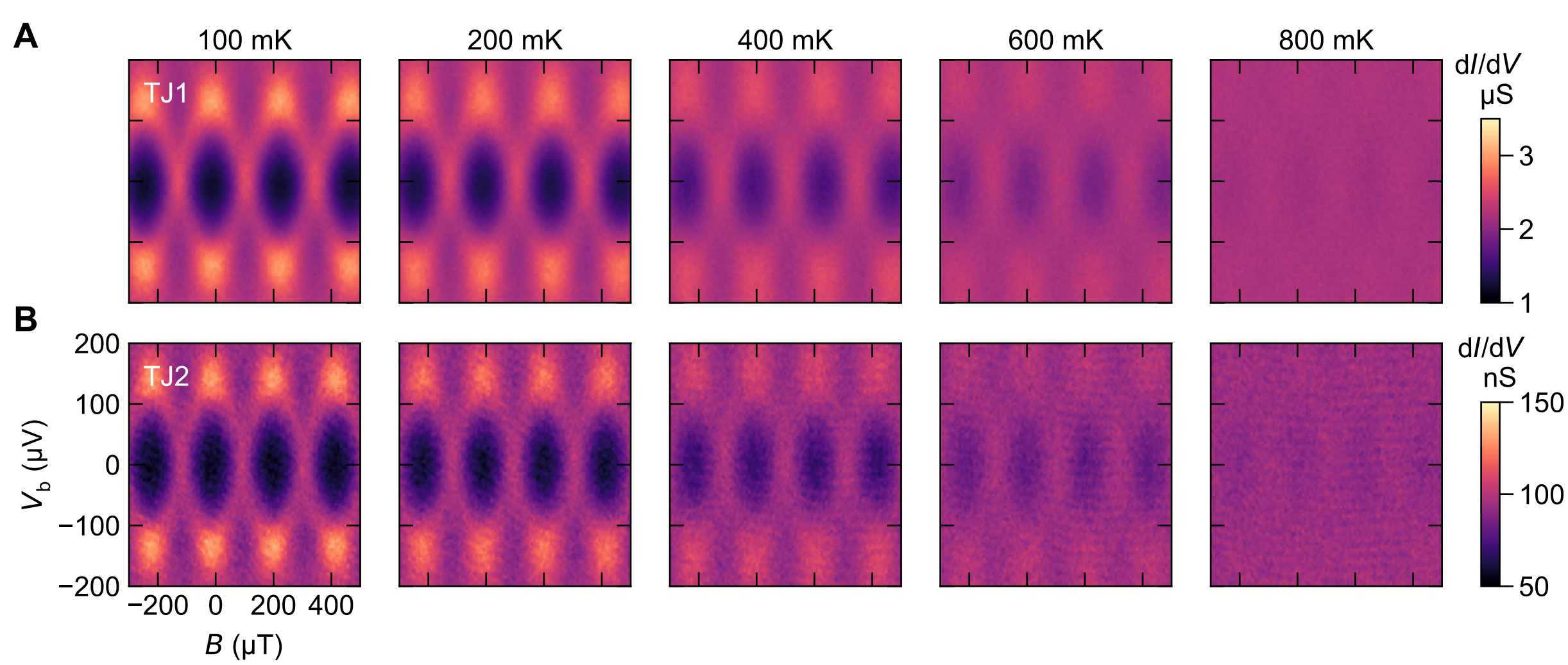

Figure S5: Evolution of the spectra for TJ1 (**A**) and TJ2 (**B**) at $V_G = 0$ with increasing temperature.

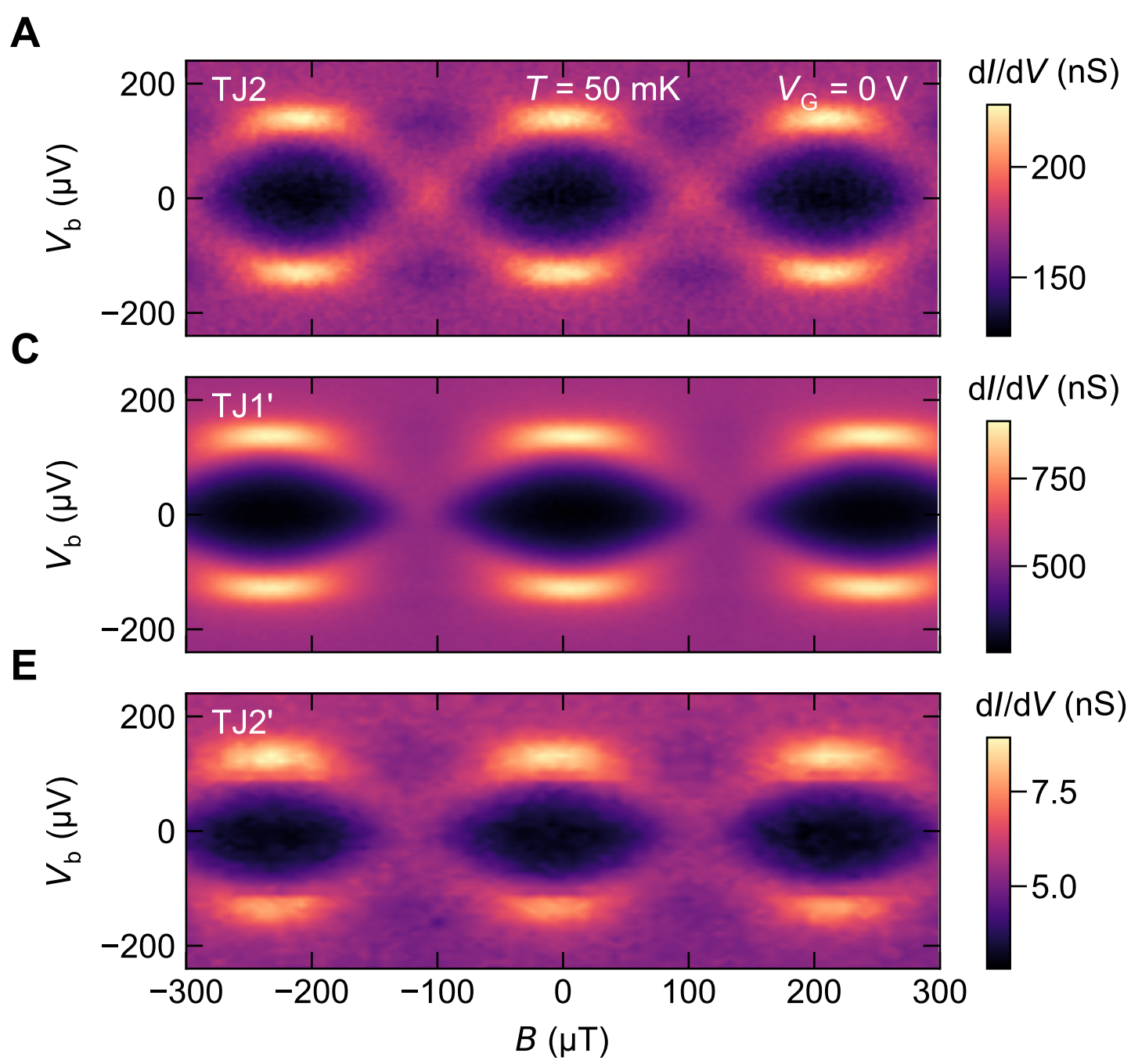

Figure S6: **A, B,**Tunnel spectra for TJ2 of the device discussed in the main text. **C-F,** Data on TJ1' and TJ2' made on an identically-designed device fabricated on a different flake of the same crystal. $V_G = 0$ V for all the data. In panels **B**, **D**, and **F**, line cuts at $\varphi = 0$ (blue) and $\varphi = \pi$ (red) are shown for each junction.

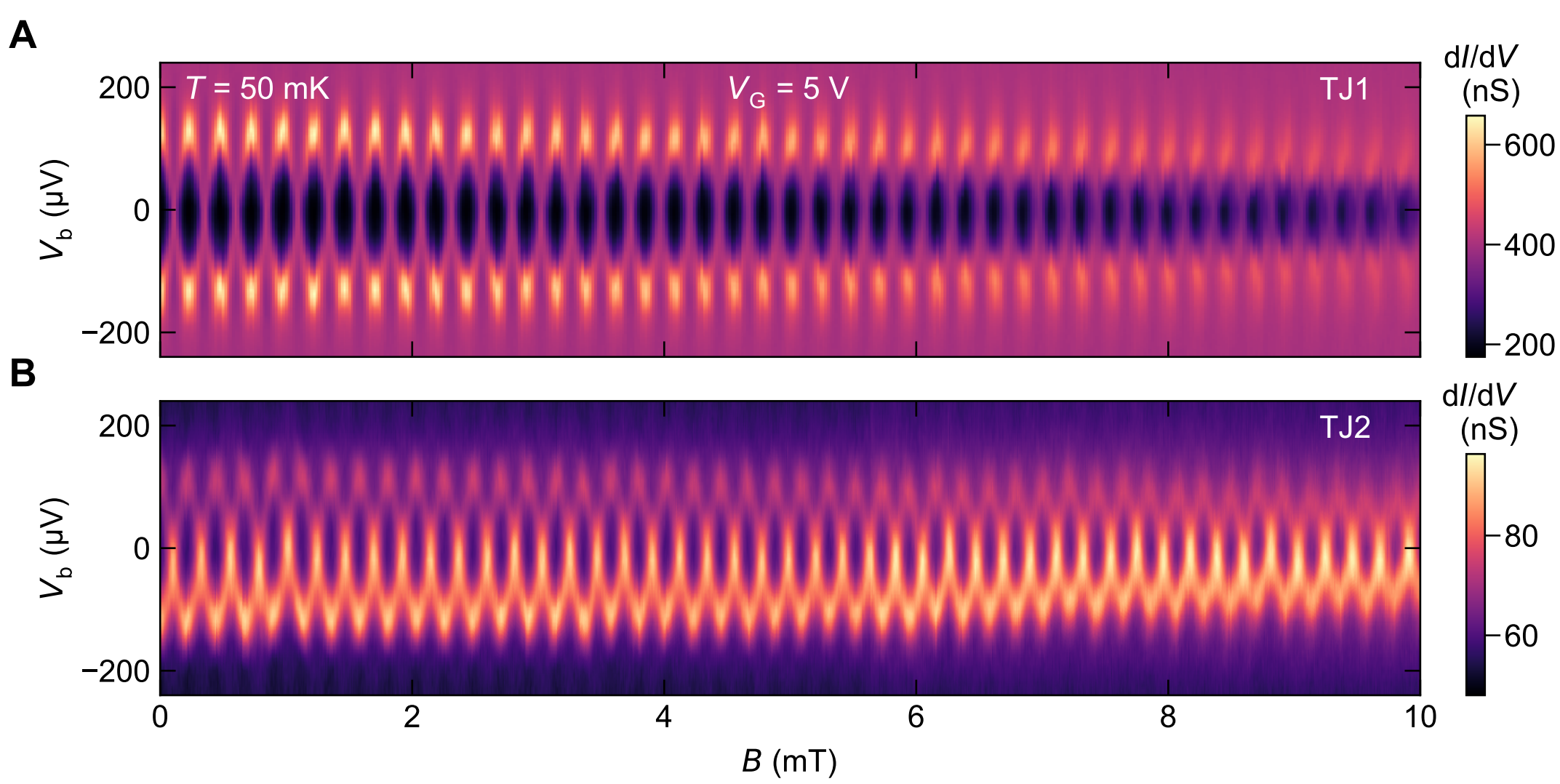

Figure S7: $\mathrm{d}I/\mathrm{d}V$ spectra measured at TJ1' (**A**) and TJ2' (**B**) for a higher magnetic field range, analogue to the data shown in Fig. S3 for device 1. The data were taken with a different current preamplifier, showing a much stronger time-dependent drift of the offset voltage that had to be numerically corrected.

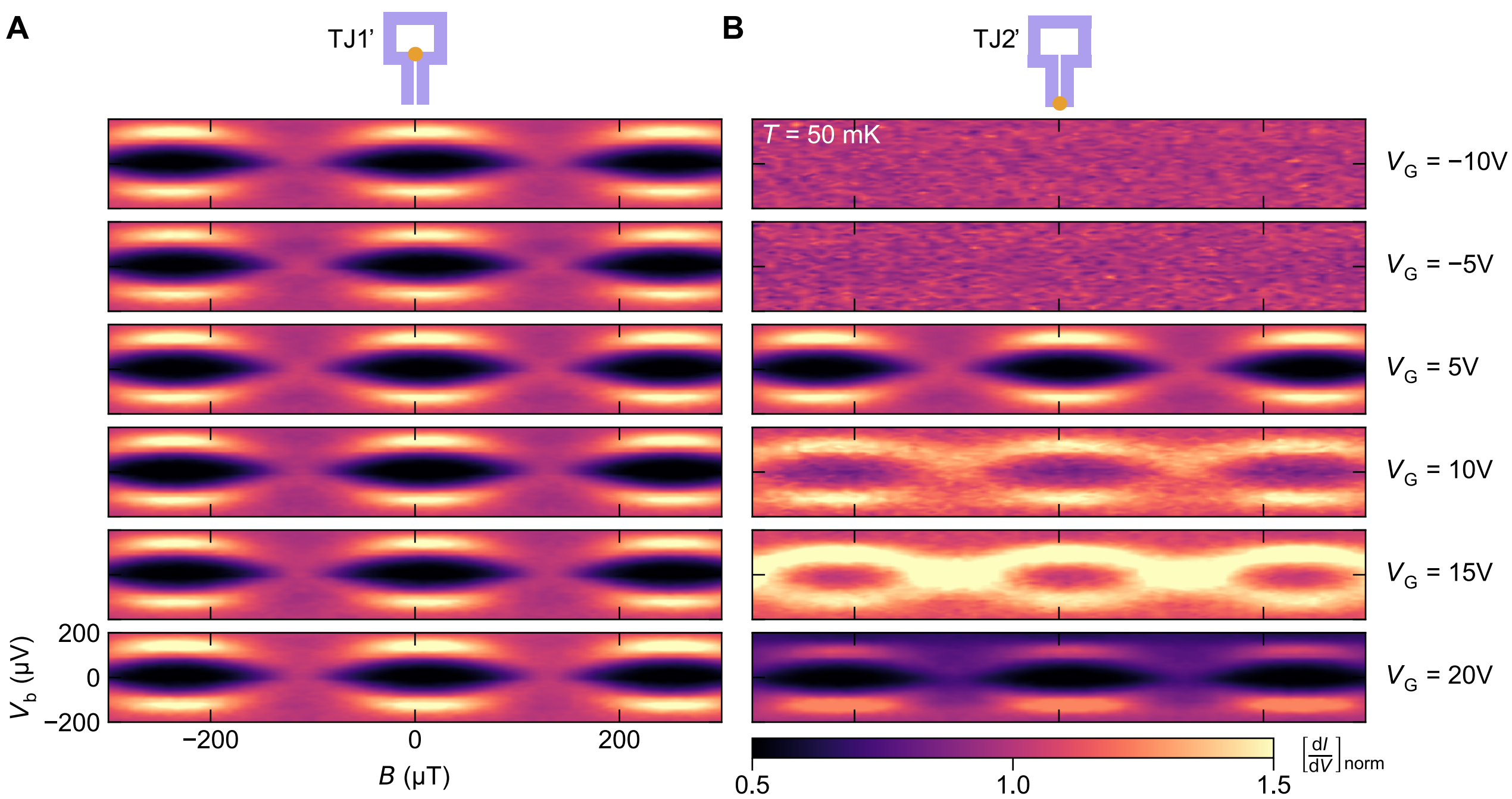

Figure S8: Spectra of TJ1' (**A**) and TJ2' (**B**) of device 2. For negative gate voltages, TJ2' becomes unmeasurable due to its conductance dropping below the noise floor.

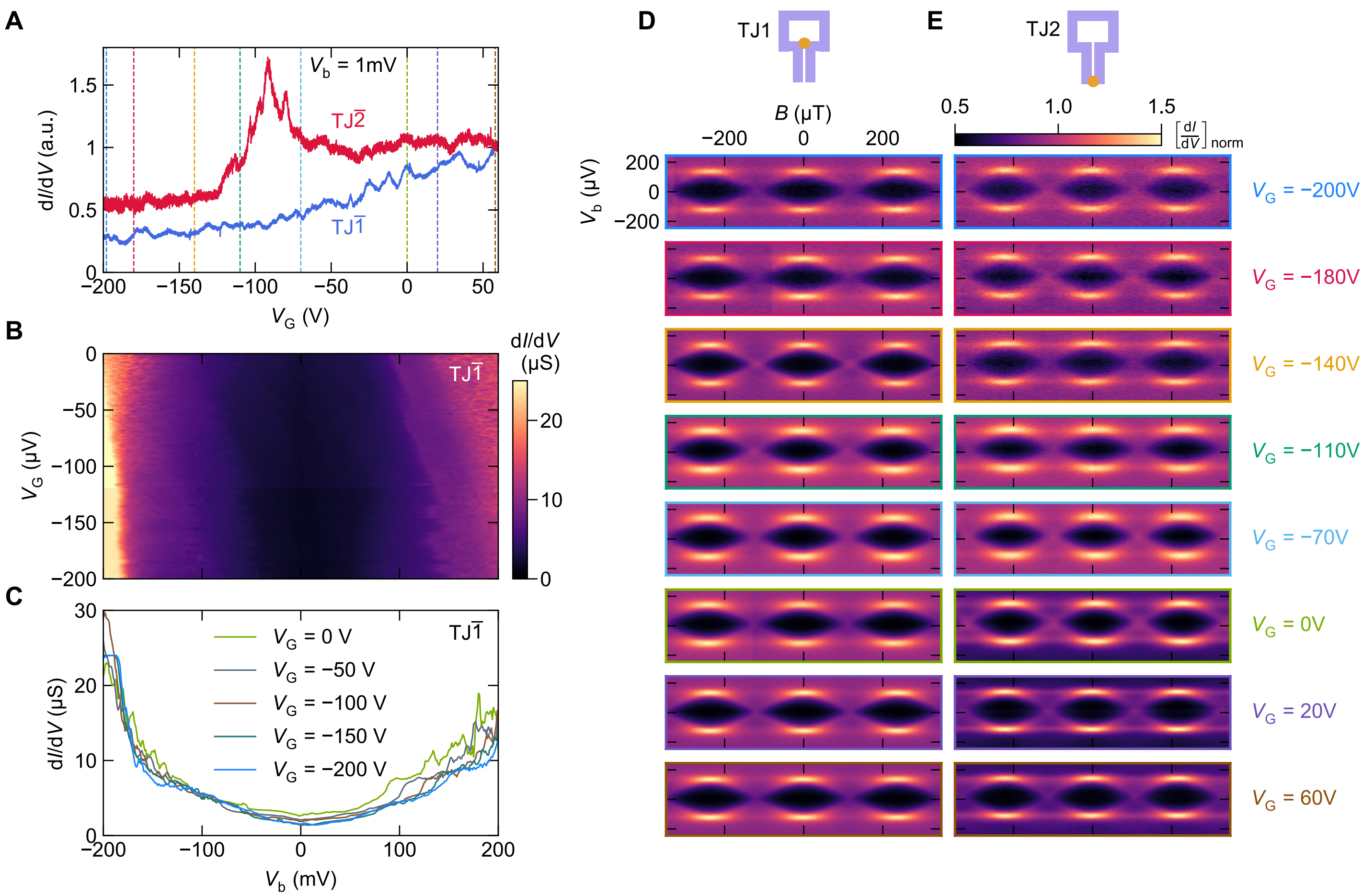

Figure S9: **Device with extended gate range. A,** Above gap differential conductance of TJ$\bar{1}$ and TJ$\bar{2}$, normalized to their respective values at 60 V. **B,** Colorplot of the differential conductance of TJ$\bar{1}$ measured over a range of bias voltages that cover the bulk band gap of the topological insulator. **C,** Line cuts of **B**, showing the systematic shift of the feature we interpret as bottom of the conduction band. **D,E,** Normalized differential conductance for TJ$\bar{1}$ and TJ$\bar{2}$, respectively, for a selection of gate voltages.

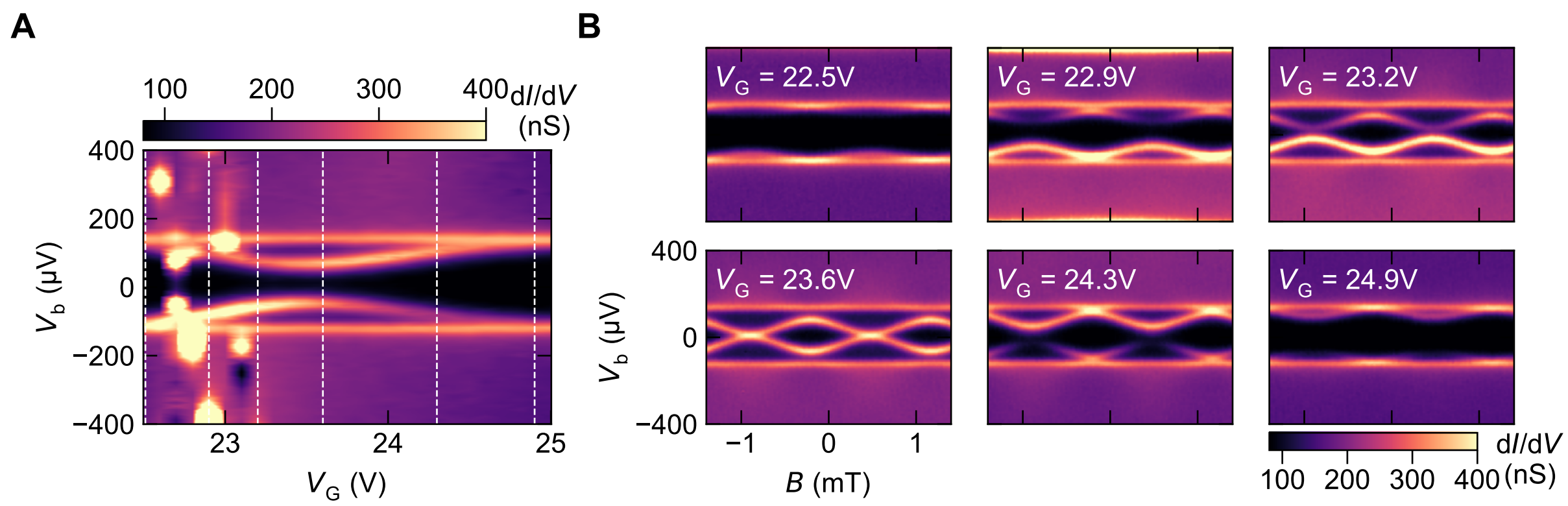

Figure S10: **Spurious resonance state. A,** $\mathrm{d}I/\mathrm{d}V$ spectra as a function of V_G and V_b measured at $\varphi = 0$ in a device similar to the one discussed in the main text. In a narrow V_G range of 22.5 – 25 V, a single ABS appeared in the SC gap. **B,** φ-dependence of the ABS spectra at selected V_G values in this narrow V_G range.

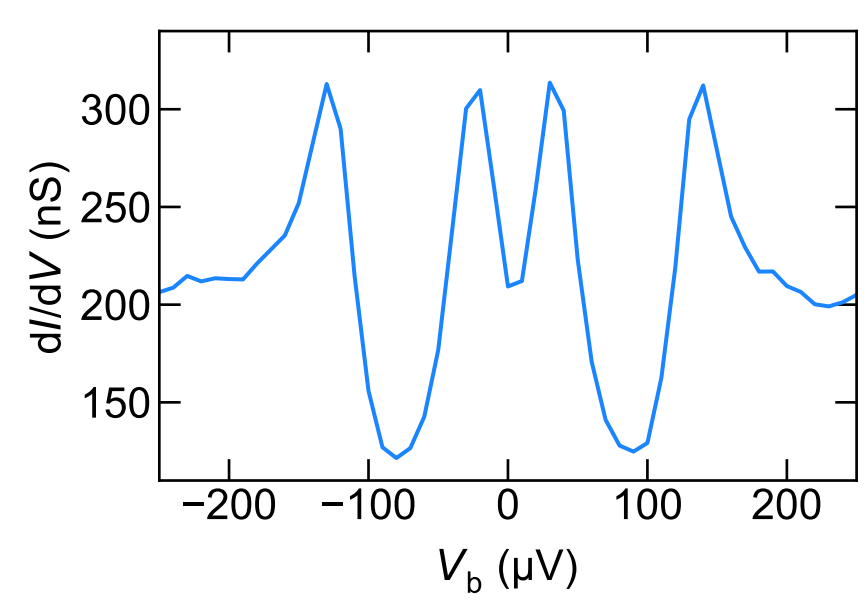

Figure S11: **Energy resolution** Line-cut of the spectrum of the Andreev quantum dot at resonance $V_G = 23.6$ V close to (but not exactly at) $\varphi = \pi$. The individual peaks located at $V_b \approx \pm 30$ µeV are clearly resolved.

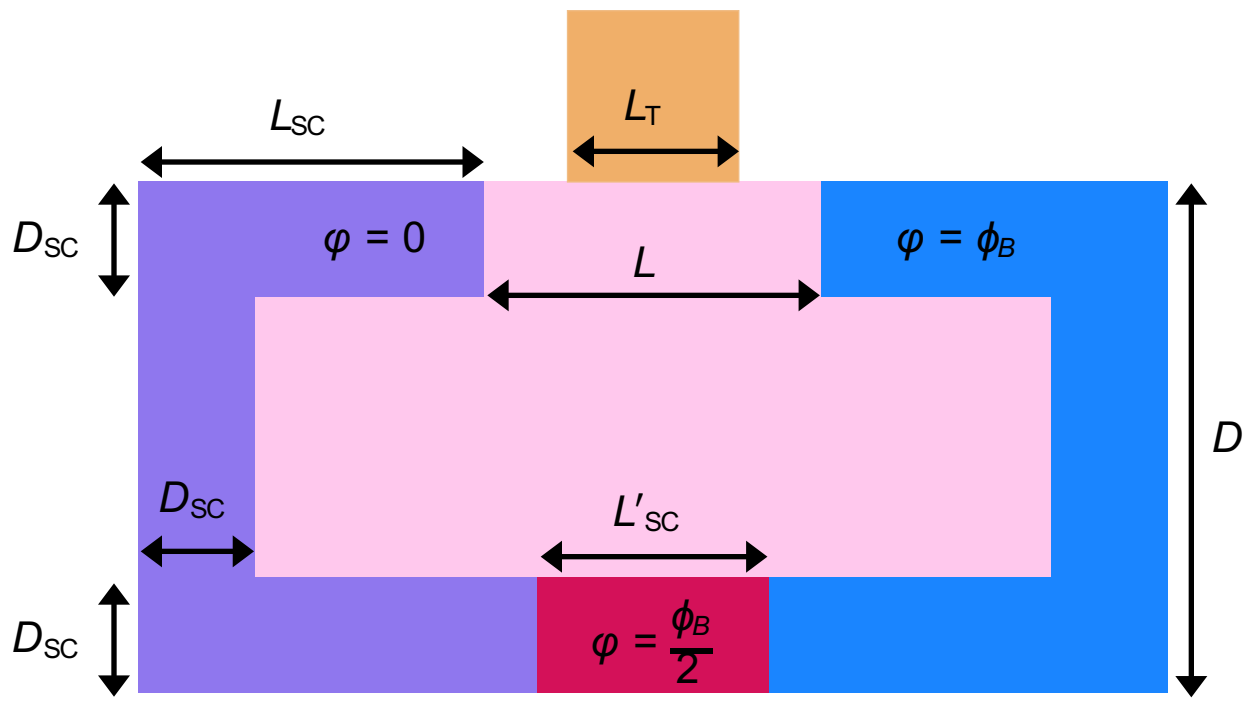

Figure S12: **Sketch of the simulated system.** The cross section of the simulated system shown with its dimensions. The bulk TI in the center (light magenta-tinted pink) is surrounded by three separate regions with finite superconducting gap Δ (lavender, magenta, azure blue). The phase of the gap function φ differs between the regions. On the top surface, this creates a Josephson junction with a finite width W and the phase difference ϕ_B. A tunnel lead (light orange) is attached in the center of the bare TI surface of the junction.

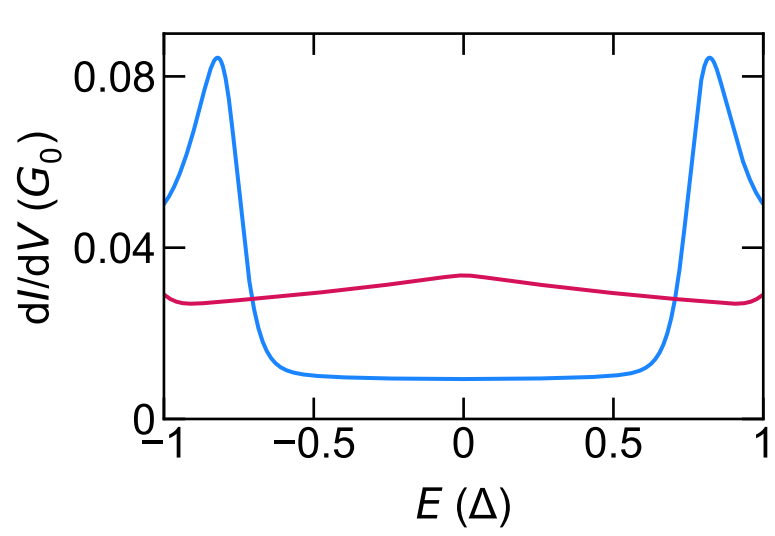

Figure S13: **Theoretical simulation without phase averaging.** This plot corresponds to Fig. 2(b) in the main text. The spectra for $\varphi = 0$ (blue) and $\varphi = \pi$ (red) were calculated without applying the running phase average. The blue line remains essentially unchanged, while the red line no longer shows a zero-bias peak.

REFERENCES

1. M. Sato, Y. Ando, Topological superconductors: A review. *Rep. Prog. Phys.* **80**, 076501 (2017).

2. A. Y. Kitaev, Unpaired Majorana fermions in quantum wires. *Phys.-Uspekhi* **44**, 131–136 (2001).

3. N. Read, D. Green, Paired states of fermions in two dimensions with breaking of parity and time-reversal symmetries and the fractional quantum Hall effect. *Phys. Rev. B* **61**, 10267–10297 (2000).

4. D. A. Ivanov, Non-abelian statistics of half-quantum vortices in *p*-wave superconductors. *Phys. Rev. Lett.* **86**, 268–271 (2001).

5. C. Nayak, S. H. Simon, A. Stern, M. Freedman, S. Das Sarma, Non-Abelian anyons and topological quantum computation. *Rev. Mod. Phys.* **80**, 1083–1159 (2008).

6. J. Alicea, New directions in the pursuit of Majorana fermions in solid state systems. *Rep. Prog. Phys.* **75**, 076501 (2012).

7. K. Flensberg, F. von Oppen, A. Stern, Engineered platforms for topological superconductivity and Majorana zero modes. *Nat. Rev. Mater.* **6**, 944–958 (2021).

8. L. Fu, C. L. Kane, Superconducting proximity effect and majorana fermions at the surface of a topological insulator. *Phys. Rev. Lett.* **100**, 096407 (2008).

9. J. R. Williams, A. J. Bestwick, P. Gallagher, S. S. Hong, Y. Cui, A. S. Bleich, J. G. Analytis, I. R. Fisher, D. Goldhaber-Gordon, Unconventional Josephson effect in hybrid superconductor-topological insulator devices. *Phys. Rev. Lett.* **109**, 056803 (2012).

10. C. Kurter, A. D. K. Finck, Y. S. Hor, D. J. van Harlingen, Evidence for an anomalous current-phase relation in topological insulator Josephson junctions. *Nat. Commun.* **6**, 7130 (2015).

11. G. Yue, C. Zhang, E. D. Huemiller, J. H. Montone, G. R. Arias, D. G. Wild, J. Y. Zhang, D. R. Hamilton, X. Yuan, X. Yao, D. Jain, J. Moon, M. Salehi, N. Koirala, S. Oh, D. J. Van Harlingen,

Signatures of Majorana bound states in the diffraction patterns of extended superconductor–topological insulator–superconductor Josephson junctions. *Phys. Rev. B* **109**, 094511 (2024).

12. J. Wiedenmann, E. Bocquillon, R. S. Deacon, S. Hartinger, O. Herrmann, T. M. Klapwijk, L. Maier, C. Ames, C. Brune, C. Gould, A. Oiwa, K. Ishibashi, S. Tarucha, H. Buhmann, L. W. Molenkamp, 4π-Periodic Josephson supercurrent in HgTe-based topological Josephson junctions. *Nat. Commun.* **7**, 10303 (2016).

13. R. S. Deacon, J. Wiedenmann, E. Bocquillon, F. Dominguez, T. M. Klapwijk, P. Leubner, C. Brune, E. M. Hankiewicz, S. Tarucha, K. Ishibashi, H. Buhmann, L. W. Molenkamp, Josephson radiation from gapless andreev bound states in HgTe-based topological junctions. *Phys. Rev. X* **7**, 021011 (2017).

14. P. Schüffelgen, D. Rosenbach, C. Li, T. W. Schmitt, M. Schleenvoigt, A. R. Jalil, S. Schmitt, J. Kölzer, M. Wang, B. Bennemann, U. Parlak, L. Kibkalo, S. Trellenkamp, T. Grap, D. Meertens, M. Luysberg, G. Mussler, E. Berenschot, N. Tas, A. A. Golubov, A. Brinkman, T. Schäpers, D. Grützmacher, Selective area growth and stencil lithography for in situ fabricated quantum devices. *Nat. Nanotechnol.* **14**, 825–831 (2019).

15. J.-P. Xu, M.-X. Wang, Z. L. Liu, J.-F. Ge, X. Yang, C. Liu, Z. A. Xu, D. Guan, C. L. Gao, D. Qian, Y. Liu, Q.-H. Wang, F.-C. Zhang, Q.-K. Xue, J.-F. Jia, Experimental detection of a majorana mode in the core of a magnetic vortex inside a topological insulator-superconductor $Bi_2Te_3/NbSe_2$ heterostructure. *Phys. Rev. Lett.* **114**, 017001 (2015).

16. P. Krogstrup, N. L. B. Ziino, W. Chang, S. M. Albrecht, M. H. Madsen, E. Johnson, J. Nygård, C. M. Marcus, T. S. Jespersen, Epitaxy of semiconductor-superconductor nanowires. *Nat. Mater.* **14**, 400–6 (2014).

17. M. Aghaee, A. Akkala, Z. Alam, R. Ali, A. Alcaraz Ramirez, M. Andrzejczuk, A. E. Antipov, P. Aseev, M. Astafev, B. Bauer, J. Becker, S. Boddapati, F. Boekhout, J. Bommer, T. Bosma, L. Bourdet, S. Boutin, P. Caroff, L. Casparis, M. Cassidy, S. Chatoor, A. W. Christensen, N. Clay, W. S. Cole, F. Corsetti, A. Cui, P. Dalampiras, A. Dokania, G. de Lange, M. de Moor, J. C. Estrada Saldaña, S. Fallahi, Z. H. Fathabad, J. Gamble, G. Gardner, D. Govender, F. Griggio, R.

Grigoryan, S. Gronin, J. Gukelberger, E. B. Hansen, S. Heedt, J. Herranz Zamorano, S. Ho, U. L. Holgaard, H. Ingerslev, L. Johansson, J. Jones, R. Kallaher, F. Karimi, T. Karzig, C. King, M. E. Kloster, C. Knapp, D. Kocon, J. Koski, P. Kostamo, P. Krogstrup, M. Kumar, T. Laeven, T. Larsen, K. Li, T. Lindemann, J. Love, R. Lutchyn, M. H. Madsen, M. Manfra, S. Markussen, E. Martinez, R. McNeil, E. Memisevic, T. Morgan, A. Mullally, C. Nayak, J. Nielsen, W. H. P. Nielsen, B. Nijholt, A. Nurmohamed, E. O'Farrell, K. Otani, S. Pauka, K. Petersson, L. Petit, D. I. Pikulin, F. Preiss, M. Quintero-Perez, M. Rajpalke, K. Rasmussen, D. Razmadze, O. Reentila, D. Reilly, R. Rouse, I. Sadovskyy, L. Sainiemi, S. Schreppler, V. Sidorkin, A. Singh, S. Singh, S. Sinha, P. Sohr, T. Stankevič, L. Stek, H. Suominen, J. Suter, V. Svidenko, S. Teicher, M. Temuerhan, N. Thiyagarajah, R. Tholapi, M. Thomas, E. Toomey, S. Upadhyay, I. Urban, S. Vaitiekėnas, K. Van Hoogdalem, D. V. Viazmitinov, S. Waddy, D. Van Woerkom, D. Vogel, J. Watson, J. Weston, G. W. Winkler, C. K. Yang, S. Yau, D. Yi, E. Yucelen, A. Webster, R. Zeisel, R. Zhao, InAs-Al hybrid devices passing the topological gap protocol. *Phys. Rev. B* **107**, 245423 (2023).

18. M. Valentini, F. Peñaranda, A. Hofmann, M. Brauns, R. Hauschild, P. Krogstrup, P. San-Jose, E. Prada, R. Aguado, G. Katsaros, Nontopological zero-bias peaks in full-shell nanowires induced by flux-tunable Andreev states. *Science* **373**, 82–88 (2021).

19. S. Das Sarma, In search of Majorana. *Nat. Phys.* **19**, 165–170 (2023).

20. R. Hess, H. F. Legg, D. Loss, J. Klinovaja, Trivial andreev band mimicking topological bulk gap reopening in the nonlocal conductance of long rashba nanowires. *Phys. Rev. Lett.* **130**, 207001 (2023).

21. A. R. Akhmerov, J. P. Dahlhaus, F. Hassler, M. Wimmer, C. W. J. Beenakker, Quantized conductance at the Majorana phase transition in a disordered superconducting wire. *Phys. Rev. Lett.* **106**, 057001 (2011).

22. T. O. Rosdahl, A. Vuik, M. Kjaergaard, A. R. Akhmerov, Andreev rectifier: A nonlocal conductance signature of topological phase transitions. *Phys. Rev. B* **97**, 045421 (2018).

23. D. I. Pikulin, B. van Heck, T. Karzig, E. A. Martinez, B. Nijholt, T. Laeven, G. W. Winkler, J. D. Watson, S. Heedt, M. Temurhan, V. Svidenko, R. M. Lutchyn, M. Thomas, G. de Lange, L. Casparis, C. Nayak, Protocol to identify a topological superconducting phase in a three-terminal device. arXiv:2103.12217 [cond-mat.mes-hall] (2021).

24. A. Banerjee, O. Lesser, M. A. Rahman, C. Thomas, T. Wang, M. J. Manfra, E. Berg, Y. Oreg, A. Stern, C. M. Marcus, Local and nonlocal transport spectroscopy in planar josephson junctions. *Phys. Rev. Lett.* **130**, 096202 (2023).

25. A. Banerjee, O. Lesser, M. A. Rahman, H.-R. Wang, M.-R. Li, A. Kringhøj, A. M. Whiticar, A. C. C. Drachmann, C. Thomas, T. Wang, M. J. Manfra, E. Berg, Y. Oreg, A. Stern, C. M. Marcus, Signatures of a topological phase transition in a planar Josephson junction. *Phys. Rev. B* **107**, 245304 (2023).

26. G. Tkachov, E. M. Hankiewicz, Helical Andreev bound states and superconducting Klein tunneling in topological insulator Josephson junctions. *Phys. Rev. B* **88**, 075401 (2013).

27. A. C. Potter, L. Fu, Anomalous supercurrent from Majorana states in topological insulator Josephson junctions. *Phys. Rev. B* **88**, 121109 (2013).

28. H. le Sueur, P. Joyez, H. Pothier, C. Urbina, D. Esteve, Phase controlled superconducting proximity effect probed by tunneling spectroscopy. *Phys. Rev. Lett.* **100**, 197002 (2008).

29. J.-D. Pillet, C. H. Quay, P. Morfin, C. Bena, A. L. Yeyati, P. Joyez, Andreev bound states in supercurrent-carrying carbon nanotubes revealed. *Nat. Phys.* **6**, 965–969 (2010).

30. L. Bretheau, J. I.-J. Wang, R. Pisoni, K. Watanabe, T. Taniguchi, P. Jarillo-Herrero, Tunnelling spectroscopy of Andreev states in graphene. *Nat. Phys.* **13**, 756–760 (2017).

31. H. Ren, F. Pientka, S. Hart, A. T. Pierce, M. Kosowsky, L. Lunczer, R. Schlereth, B. Scharf, E. M. Hankiewicz, L. W. Molenkamp, B. I. Halperin, A. Yacoby, Topological superconductivity in a phase-controlled Josephson junction. *Nature* **569**, 93–98 (2019).

32. A. Fornieri, A. M. Whiticar, F. Setiawan, E. P. Marín, A. C. C. Drachmann, A. Keselman, S. Gronin, C. Thomas, T. Wang, R. L. Kallaher, G. C. Gardner, E. Berg, E. Berg, M. J. Manfra, A. Stern, C. M. Marcus, F. Nichele, Evidence of topological superconductivity in planar Josephson junctions. *Nature* **569**, 89–92 (2019).

33. F. Nichele, E. Portolés, A. Fornieri, A. M. Whiticar, A. C. C. Drachmann, S. Gronin, T. Wang, G. C. Gardner, C. Thomas, A. T. Hatke, M. J. Manfra, C. M. Marcus, Relating Andreev bound states and supercurrents in hybrid Josephson junctions. *Phys. Rev. Lett.* **124**, 226801 (2020).

34. Z. Ren, A. A. Taskin, S. Sasaki, K. Segawa, Y. Ando, Optimizing $Bi_{2-x}SB_xTe_{3-y}Se_y$ solid solutions to approach the intrinsic topological insulator regime. *Phys. Rev. B* **84**, 165311 (2011).

35. T. Arakane, T. Sato, S. Souma, K. Kosaka, K. Nakayama, M. Komatsu, T. Takahashi, Z. Ren, K. Segawa, Y. Ando, Tunable Dirac cone in the topological insulator $Bi_{2-x}Sb_xTe_{3-y}Se_y$. *Nat. Commun.* **3**, 636 (2012).

36. S. Ghatak, O. Breunig, F. Yang, Z. Wang, A. A. Taskin, Y. Ando, Anomalous Fraunhofer patterns in gated Josephson junctions based on the bulk-insulating topological insulator $BiSbTeSe_2$. *Nano Lett.* **18**, 5124–5131 (2018).

37. C. W. J. Beenakker, Universal limit of critical-current fluctuations in mesoscopic Josephson junctions. *Phys. Rev. Lett.* **67**, 3836–3839 (1991).

38. S. Pilgram, W. Belzig, C. Bruder, Excitation spectrum of mesoscopic proximity structures. *Phys. Rev. B* **62**, 12462–12467 (2000).

39. T. Laeven, B. Nijholt, M. Wimmer, A. R. Akhmerov, Enhanced proximity effect in zigzag-shaped Majorana Josephson junctions. *Phys. Rev. Lett.* **125**, 086802 (2020).

40. I. Martin, D. Mozyrsky, Nonequilibrium theory of tunneling into a localized state in a superconductor. *Phys. Rev. B* **90**, 100508 (2014).

41. J. Stenger, T. D. Stanescu, Tunneling conductance in semiconductor-superconductor hybrid structures. *Phys. Rev. B* **96**, 214516 (2017).

42. A. Melo, C.-X. Liu, P. Rożek, T. Örn Rosdahl, M. Wimmer, Conductance asymmetries in mesoscopic superconducting devices due to finite bias. *SciPost Phys.* **10**, 037 (2021).

43. C. M. Moehle, P. K. Rout, N. A. Jainandunsing, D. Kuiri, C. T. Ke, D. Xiao, C. Thomas, M. J. Manfra, M. P. Nowak, S. Goswami, Controlling Andreev bound states with the magnetic vector potential. *Nano Lett.* **22**, 8601–8607 (2022).

44. A. Banerjee, M. Geier, M. A. Rahman, D. S. Sanchez, C. Thomas, T. Wang, M. J. Manfra, K. Flensberg, C. M. Marcus, Control of Andreev bound states using superconducting phase texture. *Phys. Rev. Lett.* **130**, 116203 (2023).

45. D. O. Oriekhov, Y. Cheipesh, C. W. J. Beenakker, Voltage staircase in a current-biased quantum-dot Josephson junction. *Phys. Rev. B* **103**, 094518 (2021).

46. F. Zhou, P. Charlat, B. Spivak, B. Pannetier, Density of states in superconductor-normal metal-superconductor junctions. *J. Low Temp. Phys.* **110**, 841–850 (1998).

47. C. W. J. Beenakker, Random-matrix theory of quantum transport. *Rev. Mod. Phys.* **69**, 731–808 (1997).

48. A. A. Taskin, Z. Ren, S. Sasaki, K. Segawa, Y. Ando, Observation of dirac holes and electrons in a topological insulator. *Phys. Rev. Lett.* **107**, 016801 (2011).

49. H. Kim, S.-J. Choi, H.-S. Sim, S. Park, Boundary-induced Majorana coupling in a planar topological Josephson junction. *Phys. Rev. B* **111**, 045414 (2025).

50. E. Nikodem, J. Schluck, H. F. Legg, M. Geier, M. Papaj, M. Bagchi, L. Fu, Y. Ando, Topological insulator nano-SQUID: Flux-tunable platform for topological superconductivity. arXiv:2412.07993 [cond-mat.mes-hall] (2025).

51. A. Murani, B. Dassonneville, A. Kasumov, J. Basset, M. Ferrier, R. Deblock, S. Guéron, H. Bouchiat, Microwave signature of topological Andreev level crossings in a bismuth-based Josephson junction. *Phys. Rev. Lett.* **122**, 076802 (2019).

52. G. Ben-Shach, A. Haim, I. Appelbaum, Y. Oreg, A. Yacoby, B. I. Halperin, Detecting Majorana modes in one-dimensional wires by charge sensing. *Phys. Rev. B* **91**, 045403 (2015).

53. S. S. Hegde, G. Yue, Y. Wang, E. Huemiller, D. J. Van Harlingen, S. Vishveshwara, A topological Josephson junction platform for creating, manipulating, and braiding Majorana bound states. *Ann. Phys.* **423**, 168326 (2020).

54. E. Grosfeld, A. Stern, Observing Majorana bound states of Josephson vortices in topological superconductors. *Proc. Natl. Acad. Sci. U.S.A.* **108**, 11810–11814 (2011).

55. C. W. Groth, M. Wimmer, A. R. Akhmerov, X. Waintal, Kwant: A software package for quantum transport. *New J. Phys.* **16**, 063065 (2014).

56. H. B. Nielsen, M. Ninomiya, The Adler-Bell-Jackiw anomaly and Weyl fermions in a crystal. *Phys. Lett. B* **130**, 389–396 (1983).

57. A. Ziesen, I. C. Fulga, F. Hassler, Geometry-independent tight-binding method for massless Dirac fermions in two dimensions. *Phys. Rev. B* **107**, 195409 (2023).

58. R. C. Dynes, V. Narayanamurti, J. P. Garno, Direct measurement of quasiparticle-lifetime broadening in a strong-coupled superconductor. *Phys. Rev. Lett.* **41**, 1509–1512 (1978).

59. W. Zhang, R. Yu, H.-J. Zhang, X. Dai, Z. Fang, First-principles studies of the three-dimensional strong topological insulators $Bi{-}2Te_3$, Bi_2Se_3 and Sb_2Te_3. *New J. Phys.* **12**, 065013 (2010).

60. D. López-Núñez, A. Torras-Coloma, Q. Portell-Montserrat, E. Bertoldo, L. Cozzolino, G. A. Ummarino, A. Zaccone, G. Rius, M. Martínez, P. Forn-Díaz, Superconducting penetration depth of aluminum thin films. *Supercond. Sci. Technol.* **38**, 095004 (2025).

61. L. F. Shatz, C. W. Christensen, Numerical inductance calculations based on first principles. *PLOS ONE* **9**, e111643 (2014).

62. J. Brede, M. Bagchi, A. Greichgauer, A. Uday, A. Bliesener, G. Lippertz, R. Yazdanpanah, A. Taskin, Y. Ando, Characterizing the chemical potential disorder in the topological insulator $(Bi_{1-x}SB_x)_2Te_3$ thin films. *Phys. Rev. Mater.* **8**, 104202 (2024).

63. A. Montag, Master's thesis, RWTH Aachen University (2023); https://doi.org/10.5281/zenodo.11615512.
